# Potent Molecular Feature-based Neutralizing Monoclonal Antibodies as Promising Therapeutics Against SARS-CoV-2 Infection

**DOI:** 10.3389/fmolb.2021.670815

**Published:** 2021-05-31

**Authors:** Arnaud John Kombe Kombe, Ayesha Zahid, Ahmed Mohammed, Ronghua Shi, Tengchuan Jin

**Affiliations:** ^1^ Department of Obstetrics and Gynecology, The First Affiliated Hospital of USTC, Division of Life Sciences and Medicine, University of Science and Technology of China, Hefei, China; ^2^ Hefei National Laboratory for Physical Sciences at Microscale, The CAS Key Laboratory of Innate Immunity and Chronic Disease, School of Basic Medical Sciences, Division of Life Sciences and Medicine, University of Science and Technology of China, Hefei, China; ^3^ CAS Center for Excellence in Molecular Cell Science, Chinese Academy of Science, Shanghai, China

**Keywords:** SARS-CoV, SARS-CoV-2, MERS-CoV, neutralizing monoclonal antibody, cross-neutralizing antibody, molecular mechanism of action, antibody cocktail

## Abstract

The 2019–2020 winter was marked by the emergence of a new coronavirus (SARS-CoV-2) related disease (COVID-19), which started in Wuhan, China. Its high human-to-human transmission ability led to a worldwide spread within few weeks and has caused substantial human loss. Mechanical antiviral control approach, drug repositioning, and use of COVID-19 convalescent plasmas (CPs) were the first line strategies utilized to mitigate the viral spread, yet insufficient. The urgent need to contain this deadly pandemic has led searchers and pharmaceutical companies to develop vaccines. However, not all vaccines manufactured are safe. Besides, an alternative and effective treatment option for such an infectious disease would include pure anti-viral neutralizing monoclonal antibodies (NmAbs), which can block the virus at specific molecular targets from entering cells by inhibiting virus-cell structural complex formation, with more safety and efficiency than the CP. Indeed, there is a lot of molecular evidence about the protector effect and the use of molecular feature-based NmAbs as promising therapeutics to contain COVID-19. Thus, from the scientific publication database screening, we here retrieved antibody-related papers and summarized the repertory of characterized NmAbs against SARS-CoV-2, their molecular neutralization mechanisms, and their immunotherapeutic pros and cons. About 500 anti-SARS-CoV-2 NmAbs, characterized through competitive binding assays and neutralization efficacy, were reported at the writing time (January 2021). All NmAbs bind respectively to SARS-CoV-2 S and exhibit high molecular neutralizing effects against wild-type and/or pseudotyped virus. Overall, we defined six NmAb groups blocking SARS-CoV-2 through different molecular neutralization mechanisms, from which five potential neutralization sites on SARS-CoV-2 S protein are described. Therefore, more efforts are needed to develop NmAbs-based cocktails to mitigate COVID-19.

## Introduction

At the end of 2019, a novel coronavirus-associated disease (COVID-19, previously 2019-nCoV), caused by an emergent coronavirus (SARS-CoV-2) (Zhu N. et al., 2020; Wu F. et al., 2020), plunged the world into dread and therefore, the scientific community to focus on the means to contain this virus. With 110,749,023 cases and 2,455,131 deaths worldwide to date (21 February 2021) https://covid19.who.int/, COVID-19 declared a pandemic by WHO on March 11, 2020 (Arabi et al., 2016), is the deadliest coronavirus pneumonia-associated disease. Additionally, because of its high potential of human-to-human transmission (R0 = 3.77 SARS-CoV-2 *Vs.* R0 = 2–3 for SARS-CoV) (Yang et al., 2020), SARS-CoV-2 spread very fast across the world, from the epicenter, Wuhan in China (Wu F. et al., 2020; Zhu N. et al., 2020), resulting in a substantial negative impact on public health and global economy. Indeed, belonging to the subgenus sarbecovirus, the *Orthocoronavirinae* subfamily, SARS-CoV-2 is the seventh member of the *Coronaviridae*'s family, which is transmitted through direct contacts with confirmed and asymptomatic COVID-19 patients, and that commonly causes fever, cough with chest tightness, respiratory distress, or dyspnea, myalgia and asthenia along with dizziness. Ground-glass opacity and patchy shadows with bilateral lesions in computed tomography analysis results, reduction in the lymphocytes accounts, and increase in C-reactive protein (CRP) accounts constitute the typical biological characteristic of COVID-19 (Li C. et al., 2020; Wu F. et al., 2020; Zhu N. et al., 2020).

Mechanical measures, including confinement, surgical mask-wearing, hand hygiene, and distancing initially imposed throughout the Chinese mainland shortly after the onset of the outbreak, and later in the rest of the affected countries, has led to a noticeable high net of improvement in the current epidemiological situation worldwide. These strategies were adopted to reduce the spread by breaking the SARS-CoV-2 transmission chain, as the scientific community lacked specific anti-coronavirus drugs. Moreover, while researches on the development of COVID-19 vaccines are ongoing, drug repositioning (use of approved antiviral drugs including Remdesivir, Favipiravir, Ribavirin, Interferons, and Lopinavir/Ritonavir) proves to be effective with an increase in the cure rate when appropriately used, although associated with some side effects. Specifically, the appropriate use of these drugs (but not chloroquine and hydro-chloroquine that are highly associated with side effects), is associated with modestly more benefits than side effects on patient healing (Jean et al., 2020; Shen et al., 2020; Beyzarov et al., 2021; Hong et al., 2021; Qaseem et al., 2021; Tian et al., 2021). For example, in China, where all these protection means were applied early and strictly, there is practically a clearance of COVID-19 (less than 600 current and 246 new cases, and 0 recent deaths) so far. Additionally, although still high, there is a considerable decrease in the incidence of infection (from 173 734, 25 540, and 31 664 in October 2020 to 28 313, 84 444, and 14 914 in February 2021) in the United States, Spain, and Italy, respectively, and in some other countries (https://covid19.who.int/). Continuing strictly adopting these protection methods/policies would overcome the current difficulties (Jin et al., 2021).

More interestingly, the current worldwide observable increase in the number of COVID-19 recovery cases also owes its success to the use of anti-SARS-CoV-2 polyclonal antibodies (pAbs) administered as plasma to acute and/or severe COVID-19 patients. Indeed, these convalescent plasmas (CPs) were obtained from volunteer recovered COVID-19 patients and have been shown to be efficient for the disease mitigations, specifically in reducing healing time and preventing death in populations at highest risk with no reported adverse effects so far (Ahn et al., 2020; Pawar et al., 2020; Rojas et al., 2020; Shen et al., 2020; Ye M. et al., 2020). This adopted empiric approach was thought from both the previous severe acute respiratory syndrome (SARS) and middle east respiratory syndrome (MERS) outbreaks in 2002 and 2012, respectively, which were contained using CPs (Cheng et al., 2005; Arabi et al., 2016). It is the neutralizing antibodies produced by the immune system, specifically the B cells (plasmocytes), in response to SARS-CoV-2 infection, which are the main characters in the mitigation of COVID-19. These glycoproteins exhibit different mechanisms to potently block the virus at any infection steps and reduce the symptoms through specific viral epitopes recognition. Therefore, scientists have engaged in researches based on the structural/molecular features of the antibody-virus immune complex to produce neutralizing antibodies specific to SARS-CoV-2. Specific neutralizing monoclonal antibodies (NmAbs) against SARS-CoV-2 and their molecular target sites have been reviewed (Chen J. et al., 2020; Raybould et al., 2020; Yuan et al., 2021). It is therefore important to gain insight into their mechanism of actions at the specific target sites for a potential antibody-based immunotherapy.

In this review, based on their structural/molecular characteristics, we summarize the specific NmAbs reported so far with the ability to block and protect against SARS-CoV-2 and their mechanisms of action at respective virus inhibition target sites. The opportunities and limits of their use in the COVID-19 eradication process are also discussed.

## Research Methodology

A comprehensive literature survey was carried out using PubMed, bioRxiv/medRxiv, Web of Science, Elsevier, Springer, Google, and Google Scholar research engines to prepare a descriptive analysis review on the current topic. The following research terms “SARS-CoV,” “SARS-CoV-2,” “SARS,” “COVID-19,” “MERS,” “MERS-CoV,” human coronavirus (HCoV) along with “Neutralizing Antibody,” “cross-neutralizing antibody,” “Convalescent plasma,” were carefully selected and randomly combined to retrieve all of the representative papers in this topic area, as for the last collection date. All original articles presenting non-characterized and non-neutralizing antibodies were excluded. Only the potently neutralizing antibodies (but not the non-neutralizing and impotent), including single-domain and complete antibodies, are reported. The SARS-CoV-2 spike glycoprotein (PDB ID: 6VYB) was retrieved and used as a model to draw and describe overall neutralization binding interfaces with hACE2 and NmAbs, at the molecular level by using PyMOL. The binding interface residues are defined based on the published antibody-antigen complex structures, describing the residues involved.

## SARS-CoV-2 NmAbs and Production Methods

Nowadays, neutralizing monoclonal and polyclonal antibodies (NmAbs and NpAbs) represent one of the best innovative therapeutic strategies to fight viral or bacterial-related infectious diseases (Prabakaran et al., 2009; Saphire et al., 2018). Since the discovery of antibodies through acquired adaptive immunity (Rojas et al., 2020), scientists have focused on the exploitation of these natural products and their effects on pathogens, resulting in the control of several pathogen-related diseases, including those caused by Nipah and Hendra viruses, and particularly the control of SARS- and MERS-CoV associated epidemics that occurred in 2002 and 2012, respectively (Traggiai et al., 2004; Prabakaran et al., 2009; Corti et al., 2015). Indeed, it is because of their functions (neutralization of the virus at the infection site, opsonization, and activation of complement) that antibodies have been considered a means to block pathogens (biological and non-biological compounds) and control pathogen-associated diseases. Moreover, their successful use against the 2002 and 2012 coronavirus (CoVs) epidemics led to the development of monoclonal antibodies (mAbs) against SARS-CoV-2 to stop the COVID-19 pandemic. For instance, it was recently shown in an animal model of SARS-CoV-2 infection that NmAbs could protect against certain SARS-CoV-2-associated damages, such as SARS-CoV-2-induced lung inflammation (Hassan et al., 2020). Additionally, plasmas from recovering or recovered COVID-19 patients, as for SARS and MERS management, have been used to treat COVID-19 patients (Duan et al., 2020; Pawar et al., 2020; Rajendran et al., 2020; Zeng Q. L. et al., 2020), suggesting the huge importance of antibodies in COVID-19 mitigation. Therefore, numerous studies have been carried out to isolate antibodies from COVID-19 CPs, as large-scale plasma collection and associated risks to treat the worldwide-infected population constitute one of the limitations of the direct use of CPs (Bloch et al., 2020). It is noteworthy that numerous NmAbs have also been isolated from healthy people and survivor SARS patients, as reports demonstrated evidence of cross-mAbs against SARS-CoV-2 (Lv H. et al., 2020; Walls et al., 2020).

Besides human plasmas, other sources have been used to produce NmAbs against SARS-CoV-2, including animals (alpaca, camel, equine, llama, and mouse) and synthetic sources.

The methods adopted to develop mAbs against SARS-CoV-2 range from empirical to recently developed innovative methods (BalcioGlu et al., 2020). Empirical methods used to produce anti-SARS-CoV-2 antibodies include ribosome, phage, yeast, and retained display technologies (Beasley et al., 2020; Li T. et al., 2020; Wu et al., 2020a), allowing to produce more than hundreds of antibodies. Phage and yeast display-based mAbs development technologies have subsequently been improved for fast isolation of antibodies (Wu et al., 2020b; Zost et al., 2020b). On the other hand, technology innovation has allowed the development of new, simpler, and less time-consuming antibody production methods. For instance, the possibility of better isolating potent human NmAbs (hNmAbs) lies in the technological innovation-based ability to sort single B cells from human plasma sources and clone the antibody sequences for selecting the potent neutralizers (Traggiai et al., 2004; Smith et al., 2009; Huang et al., 2013; Smith et al., 2014). Besides, techniques such as Machine Learning technologies (Magar et al., 2020), in the context of the fight against SARS-CoV-2, made it possible to produce numerous mAbs. In addition, single-cell sequencing, CDR3_H_ structure similarity, high-efficiency screening, mutation-based maturation and engineering, and synthetic approaches (Custódio et al., 2020; Han et al., 2020; Li T. et al., 2020; Miersch et al., 2020; Rouet et al., 2020; Schoof et al., 2020) or approaches from genetically humanized mice led to the production of mAbs with well-proven potency. Furthermore, the use of proteomics, an innovative approach in sorting neutralizing immunoglobulins in hilled COVID-19 patients allowed Voss *et al.* to develop outstanding neutralizing antibodies with high ability to protect efficiently against experimental SARS-CoV-2 infection (Voss et al., 2020). Recently, an amazing technology allowing the development of highly potent Fc fragment combination-based multivalent antibodies set-up by Rujas et al. (Rujas et al., 2020) as well as a synthetic recombinant antibody generation technology that mimic *in vivo* somatic hypermutations, referred to as Autonomous Hypermutation yEast surfAce Display (AHEAD) set-up by Wellner et al. (Wellner et al., 2020) are added to the above technologies to contain the COVID-19.

## SARS-CoV-2 NmAb Molecular Neutralizing Activities

### SARS- and MERS-CoV NmAbs with Cross-Activities

As with all CoVs, the SARS-CoV-2 envelope glycoprotein (spike protein) is the primary protein involved in the process of virus attachment and integration into the target host cell during infection. Determination of its structure revealed several domains—known as functional structural domains—involved in attachment and fusion to epithelial cells. Briefly, the CoVs S protein (∼1,273 a. a.) consists of two subunits, including S1 and S2, which constitute the extracellular domain, and within which there are antigenic domains, the more important of which so far are RBD and HR1/HR2, respectively. Besides, transmembrane and intracellular domains in the C-terminal region are not involved in virus-cell interactions. During the infection process, human coronavirus (HCoVs), especially SARS-CoV-2, mediated by S protein, firstly bind to gangliosides (Fantini et al., 2021), then simultaneously to heparan sulfate (HS) and hACE-2 through recognition by the viral surface antigens, RBD (aa 330–583, for the SARS-CoV-2 RBD) (Clausen et al., 2020); Lan et al., 2020). Subsequently, the virus fuses with the cell membrane through S2 (HR1 and HR2) before being internalized by endocytosis (Ou et al., 2020; Tai et al., 2020a). The spike protein is then mainly responsible for triggering the development of the adaptive response.

First, because the SARS-CoV-2 S protein has structural homologies with that of SARS-CoV (∼70% and 86% sequence identity and similarity with SARS-CoV RBD, respectively (Wan Y. et al., 2020; Zhou P. et al., 2020)) and MERS-CoV, the hypothesis of a selective genetic evolution or genetic drift in the *Coronaviridea* family is supported instead of gene manipulation or laboratory construct, as suspected (Andersen et al., 2020). So, these sequence similarities between RBD-SARS-CoV and RBD-SARS-CoV-2 led researchers to hypothesized and then later to demonstrate that the SARS-CoV-2 S attaches to cellular receptors in the same way as SARS-CoV S does (Ceccarelli et al., 2020; Tai et al., 2020; Tian et al., 2020; Wan Y. et al., 2020), and thus to explore the activity of the known SARS-CoV specific NmAbs, on SARS-CoV-2, pending a cross-activity. By focusing primarily on antibodies targeting conserved epitopes between these two sarbecoviruses, potential cross-neutralizing activity was expected (Tai et al., 2020b; Tian et al., 2020). Previous reports have shown that while CPs from SARS-patients can cross-react with and cross-neutralize SARS-CoV-2 virus (Lv H. et al., 2020), polyclonal antibodies obtained from mice immunized with purified SARS-CoV S, exhibit a cell protection against SARS-CoV-2 entry, suggesting the existence of cross-NmAbs (Walls et al., 2020).

From these studies, it was reported that several SARS-CoV-specific NmAbs from different sources, targeting conserved epitopes, could potently cross-react with and cross-neutralize SARS-CoV-2 (Table 1). Specifically, screening B lymphocytes repertory isolated from patient blood of SARS survivors enabled isolation of 200 antibodies, including 153 which cross-react with SARS-CoV-2 S, but only 8, including ADI-55689, ADI-55993, ADI-56000; ADI-55688, ADI-56046, ADI-56010, ADI-55690, and ADI-55951 could potently cross-neutralize SARS-CoV-2 (Wec et al., 2020). Together with the nanobody VHH-72, isolated from llama (Wrapp et al., 2020a), these NmAbs target conserved epitopes between SARS-CoV and SARS-CoV-2, as expected. More interestingly, the conserved epitopes targeted by these cited cross-neutralizers overlap the ACE2-binding site on SARS-CoV-2 RBD. For instance, consistent with the competitive ELISA binding assay results that revealed a strong competition of VHH-72, ADI-55689, ADI-55993, ADI-56000, and ADI-55688 with ACE2 to bind RBD, structural analysis of some of these bounds with SARS-CoV-2 RBD revealed that they only bury a unique region that is same as that of ACE2. This suggests that these five (Li C. et al., 2020) potent NmAbs exhibit the same unique neutralizing/inhibition mechanism against authentic or wild-type (WT) and/or pseudotyped (PT) SARS-CoV-2, which consist of the direct blocking of SARS-CoV-2 to bind to ACE2. Better than these previous ones, ADI-56046, ADI-56010, ADI-55690, and ADI-55951, with VHH-72, can potently prevent WT and PT SARS-CoV-2 entry into cells through a twofold binding at two distinct epitopes including ACE2 and CR3022-specific epitopes, as they can compete with ACE2 and CR3022, respectively. This suggests a supplementary neutralization mechanism to the direct ACE2-binding inhibition, which could be a steric hindrance (Wec et al., 2020; Wrapp et al., 2020a). The efficacy of VHH-72 is enhanced when fused with the constant region Fc as a bivalent human IgG (VHH-72-Fc). Unlike CR3022, VHH-72 can cross-react with and cross-neutralize SARS-CoV-2 by targeting the cryptic epitope because, as a nanobody, VHH7-72 is smaller and less susceptible to steric hindrance than the full antibody CR3022 (Forsman et al., 2008).

**TABLE 1:** SARS- and MERS-CoV-specific NmAbs with cross-neutralizing activity against SARS-CoV-2.

Antibody	Source	Targeted site on SARS-CoV-2	Cross-neutralizing activity	Reported neutralization mechanism	IC_50_ (µg/ml)	Ref
S309 and its Fab, VIR-7831	Human	RBD (distinct from ACE2 binding site)	Cross-neutralize PT and WT SARS-CoV-2 infection	Independent of hACE2-binding inhibition, and hypothetically through either steric hindrance or S trimer cross-linking	0.79	Pinto et al. (2020a); Pinto et al. (2020b)
ADI-55689, ADI-55993, ADI-56000; ADI-55688	Human	RBD (overlapped ACE2-binding site)	Cross-neutralize PT and WT SARS-CoV-2 infection	Prevent S RBD binding to receptor via direct ACE2-binding inhibition, and exhibit a neutralization via other factors beyond binding affinity	NA	Wec et al. (2020)
						
ADI-56046, ADI-56010, ADI-55690, ADI-55951	Human	RBD (overlap ACE2- and CR3022-binding site)	Cross-neutralize PT and WT SARS-CoV-2 infection	Prevent S RBD binding to receptor via direct ACE2-binding inhibition	NA	Wec et al. (2020)
						
S304 and S315	Human	RBD	Cross-neutralize PT, exhibits synergic neutralization with S309 against PT and WT SARS-CoV-2	Recognize the target with a weak affinity.	NA	Pinto et al. (2020a); Pinto et al. (2020b)
CR3022-B6 CR3022-M	Human	RBD (distinct from ACE2-binding site)	Neutralize PT SARS-CoV-2 infection	Hamper RBD-ACE2 binding	0.35; 4.4	Rouet et al. (2020)
						
m396-B10; m396-C4	Human	RBD (distinct from ACE2-binding site)	Both neutralize WT, and m396-B10 neutralizes PT	Hamper RBD-ACE2 binding	0.16; 0.34	Rouet et al. (2020)
						
80R-A2	Human	RBD (distinct from ACE2-binding site)	Both neutralize WT, and m396-B10 neutralizes PT	Hamper RBD-ACE2 binding	17.8	Rouet et al. (2020)
VHH-72	Llama	RBD (overlapped ACE2-binding site)	Cross-neutralize pseudoviruses and wild-type SARS-CoV-2 infection	Prevent S binding to receptor via direct ACE2-binding inhibition	0.2	Wrapp et al. (2020a)
18F3	Mouse	RBD (distinct from ACE2-binding site)	Cross-neutralize pseudoviruses and wild-type SARS-CoV-2 infection	Independent mechanism of hACE2-binding inhibition	10	Tai et al. (2020b)
7B11	Mouse	RBD (close, but distinct from ACE2-binding site)	Cross-neutralize PT and WT SARS-CoV-2 infection	Blocking SARS-CoV-2 binding to ACE2	10	Tai et al. (2020b)
154C, 240C	Mouse	S	Cross-neutralize SARS-CoV-2 infection	Prevent S binding to the receptor		Bates et al. (2020)
B6	Mouse	S, RBD	MERS- and SARS-CoV Ab, Cross-neutralizes WT SARS-CoV-2	Direct ACE2-binding inhibition	0.06	Sauer et al. (2021)

NA, Non-attributed.

Besides, 18F3, 7B11, S309, and its Fab, S304, S315, 154C, and 240C are other SARS-CoV-specific neutralizing antibodies that potently cross-neutralize SARS-CoV-2 by targeting conserved or partially-conserved (for 7B11) epitopes that are distinct from the receptor-binding motif (RBM), responsible for virus attachment to ACE2. For instance, ELISA assays showed no competitive effect between each of these non-RBM cross-NmAbs and ACE2 to bind to SARS-CoV-2 RBD (Tai et al., 2020b; Tian et al., 2020; Yuan M. et al., 2020). This suggests that the inhibition mechanism differs from the direct interference of SARS-CoV-2 RBM to bind ACE2 but rather is a steric hindrance-based inhibition mechanism, preventing WT and/or PT SARS-CoV-2 to infect cells that express hACE2 protein (Bates et al., 2020; Pinto et al., 2020a; Pinto et al., 2020b; Tai et al., 2020b).

On the other hand, it has been reported many SARS-CoV specific NmAbs with cross-reactivity against but failed to neutralize SARS-CoV-2, among which CR3022, m396, CR3014, and 80R. Rouet et al. (Rouet et al., 2020) demonstrated that mutation-based maturation and engineering into those cited NmAb sequences led to the new antibody variants with the ability to neutralize SARS-CoV-2. For instance, NmAb variants CR3022-B6, CR3022-M, m396-B10, m396-C4, and 80R-A2 displayed a moderate to high binding affinity to RBD of 290, 188, 7.1, 13, and 61 nM, respectively, correlating with highly potential SARS-CoV-2 neutralization (Table 1). No CR3014-related mutants were able to neutralize SARS-CoV-2 efficiently.

When it comes to MERS-CoV-specific NmAbs with cross-neutralizing activity against SARS-CoV-2, aside from Chouchane et al. (Chouchane et al., 2021) who recently identified MERS-CoV-specific pAbs from non-immunized camel, with SARS-CoV-2 cross neutralization, but without further characterization, only one SARS- and MERS-CoV specific antibody, namely B6 was able to cross-neutralize SARS-CoV-2 in nanomolar concentration range (Sauer et al., 2021).

All these reported SARS-CoV-specific cross-neutralizers target conserved epitope on SARS-CoV-2 RBD and exhibit either an inhibition mechanism directly dependent on ACE2-binding inhibition or a steric hindrance-based inhibition. So far, no SARS-CoV-specific NmAbs targeting sites other than S1_B_ have been reported to cross-neutralize SARS-COV-2.

### Other HCoVs NmAbs with Cross-Activities

Two cases of SARS-CoV-2 antibody isolation studies demonstrated unexpected but interesting results. Firstly, among the 200 antibodies that Wec *et al.* (Wec et al., 2020) isolated from SARS-patient survivor blood, a set of uncharacterized antibodies were surprisingly not able to bind SARS-CoV, but only specific to SARS-CoV-2. Moreover, they confirmed that these mAbs were not SARS-CoV-specific antibodies after isolating other panels of antibodies reacting with SARS-CoV-2 from three healthy adult donors with serological evidence of circulating HCoV exposure and no history of SARS-CoV or SARS-CoV-2 infection. Secondly, Bertoglio et al. (Bertoglio et al., 2020) also isolated 17 proven SARS-CoV-2-specific NmAbs with high protective efficacy against WT infection from pre-pandemic healthy donors (Table 2). All the 17 NmAbs recognize isolated RBD, and most of them, but not all, overlap the ACE2 binding site on SARS-CoV-2 S1, and as they exhibit a synergic neutralization effect, they revealed not to target the same epitopes on RBD. For instance, antibodies that target epitopes overlapping the ACE2-binding site, specially STE72-8A2, STE72-8A6, STE72-8E1, STE73-2B2, STE73-6C1, and STE73-6C8, exhibit direct prevention of RBD and S to bind to ACE2. However, antibodies targeting epitopes away from ACE2 epitope, like STE73-2C2 and STE73-2G8, exhibit a steric hindrance either through conformational changes or by preventing the RBD from adopting the required "open" conformation for ACE2 binding on the spike full length. These observations suggest that they can be used in the same cocktail as immunotherapeutic.

**TABLE 2:** Reported healthy people-specific NmAbs with neutralizing activity against SARS-CoV-2.

Ab name	Target site on SARS-CoV-2	Cross-neutralizing activity	Reported neutralization mechanism	IC_50_
STE72-8A2,				0.3
STE72-8A6,				0.56
STE72-8E1,	RBD (overlapped ACE2-binding site)	Cross-neutralize wild-type SARS-CoV-2 infection	Prevent S binding to receptor via direct ACE2-binding inhibition	1.1
STE73-2B2,				0.4
STE73-6C1,				0.81
STE73-6C8				1.08
STE73-2C2,	RBD (distinct from ACE2-binding site)	Cross-neutralize wild-type SARS-CoV-2 infection	Either steric hindrance or conformation changes	0.4
STE73-2G8				0.6
STE70-1E12,				0.64
STE72-1B6,				0.96
STE72-1G5,				NA
STE72-4C10				0.7
STE72-2G4,	RBD	Cross-neutralize wild-type SARS-CoV-2 infection	Prevent S binding to receptor	0.6
STE73-2E9,				0.74
STE73-6B10				0.68
STE73-9G3,				14.6
STE72-4E12				0.6

The IC_50_ (nM) was measured using 10 nM RBD and ACE2 positive cells (Bertoglio et al., 2020).

To explain these unexpected results, Wec et al. (Wec et al., 2020) suggested that most of these NmAbs result from preexisting memory B cells induced through infection by other circulating HCoVs strains, as HCoV S proteins resemble up to 32% the SARS-CoV and SARS-CoV-2 S proteins. Therefore, new infection by SARS-CoV triggers these RBD-specific memory B cells. Furthermore, inherent differences in the stability or antigenicity between SARS-CoV-2 A and SARS-CoV S (Wec et al., 2020), and existence of closer conserved epitopes between SARS-CoV-2 and other sarbecovirus other than SARS-CoV, explain why certain NmAbs don’t recognize SARS-CoV S. Beside, because of the different infectivity, these HCoVs-specific antibodies bind to SARS-CoV-2 S rather than SARS-CoV S, as SARS‐CoV‐2 S binds to hACE2 with higher affinity than SARS‐CoV S protein does (10- to 20-folder) (Wrapp et al., 2020b).

### Human and Humanized SARS-CoV-2 NmAbs

Several SARS-CoV–specific NmAbs were expected to exhibit a cross-neutralization activity against SARS-CoV-2 potently, as certain exhibit a strong cross-reactivity and/or others recognize conserved epitopes between both SARS-CoV S and SARS-CoV-2 S (Tian et al., 2020). Unfortunately, only a few presented hereinbefore (Tables 1, 2) targeting anywhere else on S RBD showed a cross-neutralizing activity consistent with the cross-binding ability. Therefore, knowing COVID-19 as a highly infectious disease within the beta-coronavirus family, the development of specific NmAbs was carried out and resulted in satisfying outcomes.

Among the SARS-CoV-2–specific NmAbs, the full antibodies obtained from human sources (COVID-19 infected or convalescent patients) account for more than 70% of reported antibodies, and most of them were obtained from enriched sorted PBMCs (Table 3).

**TABLE 3:** Reported SARS-CoV-2-specific NmAbs.

Antibody	Isolation method (source)	Targeted epitope (residues)	Neutralizing activity and protection efficacy	Neutralization mechanism	K_D_ (nM)	IC_50_	Ref
0304-3H3; 9A1	PBMC (H)	S, S2	0304-3H3 and 9A1 neutralize WT and PT SARS-CoV-2, respectively	Block the viral fusion to cell membrane	4.52; <10^–3^	0.04; ---	Chi et al. (2020b)
1–20; 1–57; 2–4; 2–7; 2–15, 2–36; 4–20	PBMC (H)	RBD, RBM (overlap ACE2-binding site)	Neutralize WT and PT SARS-CoV-2 infections	Direct ACE2-binding inhibition	NA	0.0007–0.209 and 0.005–0.512	Liu L. et al. (2020)
							
1–68; 1–87; 2–17; 4–8; 4–18; 4–32; 4–19; 5–24	PBMC (H)	NTD	Neutralize WT and PT SARS-CoV-2 infections	Block the first steps of membrane attachment	NA	0.007–0.109 and 0.013–0.767	Liu L. et al. (2020)
							
1F4; 2H4; 6F8; 3F11	PBMC (H)	RBD	Neutralize WT and PT SARS-CoV-2 infections	Direct ACE2-binding inhibition	1.6–120	1.6–4.5; 0.1–0.9	Wang B. et al. (2020)
1M-1D2	PBMC (H)	S/RBD, compete with 4A8	Neutralize WT SARS-CoV-2 *in vitro*	Direct ACE2-binding inhibition	108	28.0	Chi et al. (2020b)
206 Abs^a^ (P2C-1F11; P2B-2F6; P2C-1A3)	PBMC (H)	RBD (P2B-2F6 [K444- S494] partially overlap ACE2-binding site)	All neutralize WT and PT SARS-CoV-2 infections	hACE2-binding inhibition through steric hindrance	1.3–21.29	(0.03; 0.05; 0.62)	Ju et al. (2020)
2–30; 2–38	PBMC (H)	RBD	Neutralize WT and PT SARS-CoV-2 infections	Indirect ACE2-binding inhibition	NA	0.05; 0.512 and 0.208, 0.232	Liu L. et al. (2020)
2–43	PBMC (H)	S/RBD (tertiary structure)	Neutralize WT and PT SARS-CoV-2 infections	hACE2-binding inhibition through probable steric hindrance	NA	0.003; 0.071 and 0.007; 0.652	Liu L. et al. (2020)
2–51	PBMC (H)	S/NTD (tertiary structure)	Neutralize WT and PT SARS-CoV-2 infections	Block the first steps of membrane attachment	NA	0.003; 0.071 and 0.007; 0.652	Liu L. et al. (2020)
2M-10B11	PBMC (H)	RBD (distinct from ACE2-binding site)	Neutralize PS SARS-CoV-2 infection	Independent of hACE2-binding inhibition	NA	170	Chi et al. (2020b)
311mab–31B5; 311mab–32D4	PBMC (H)	RBD	Neutralize PS SARS-CoV-2 infection	Direct ACE2-binding inhibition	NA	0.0338, 0.0698	Chen X. et al. (2020)
413–2; 505–8	PBMC (H)	RBD/S (tertiary structure)	Neutralize WT and PT SARS-CoV-2 infections	Direct ACE2-binding inhibition	0.06; 17.62	1.40; 0.056	Wan J. et al. (2020)
414–1; 515–5; 553–15; 553–60,63; 505–1,3,5	PBMC (H)	RBD	Neutralize WT and PT SARS-CoV-2 infections	Direct ACE2-binding inhibition	0.31–119.7	0.02–1.40	Wan J. et al. (2020)
4A8	PBMC (H)	NTD (loops 141–156, 246–260), and S1/S2 cleavage site	Neutralize WT and PT SARS-CoV-2 infections	ACE2-binding inhibition through Conformational change	92.7	0.61and 49.0	Chi et al. (2020b); Cheng et al. (2020)
553–49	PBMC (H)	RBD	Neutralize PT SARS-CoV-2 infections	Direct ACE2-binding inhibition	0.076	0.014	Wan J. et al. (2020)
5A6	Phage display (H)	RBD	Neutralize WT and PT SARS-CoV-2 infections	Bivalent hACE2-binding inhibition	7.61	0.07 and 0.005	Wang B. et al. (2020)
5C2; 1G6; 1C10	Phage display (H)	S/RBD	Neutralize WT SARS-CoV-2 infections	Indirect ACE2-binding inhibition	NA		Yuan A. Q. et al. (2020)
B38	PBMC (H)	RBD (36 a.a. from R403 to Y505)	Neutralize WT SARS-CoV-2 infection; synergic neutralization with H4	Direct hACE2-binding inhibition	78.1	0.177	Wu et al. (2020c)
B5	PBMC (H)	RBD	Neutralize WT SARS-CoV-2 infection	Partial competition with hACE2	305	1.375	Wu et al. (2020c)
BD-217	Single-cell sequencing (H)	S (RBD)	Neutralize WT and PT SARS-CoV-2 infections	Direct ACE2-binding inhibition (compete with ACE2)	0.29	0.84 and 0.031	Cao et al. (2020)
BD-218	Single-cell sequencing (H)	S (RBD)	Neutralize WT and PT SARS-CoV-2 infections	Direct ACE2-binding inhibition (compete with ACE2)	0.039	0.29 and 0.011	Cao et al. (2020)
BD-236	Single-cell sequencing (H)	S (RBD)	Neutralize WT and PT SARS-CoV-2 infections	Direct ACE2-binding inhibition (compete with ACE2)	2.8	0.52 and 0.037	Cao et al. (2020)
BD-361	Single-cell sequencing (H)	RBD	Neutralize WT and PT SARS-CoV-2 infections	Direct ACE2-binding inhibition (compete with ACE2)	1.3	0.78 and 0.02	Cao et al. (2020)
BD-368	Single-cell sequencing (H)	RBD	Neutralize WT and PT SARS-CoV-2 infections	Direct ACE2-binding inhibition (compete with ACE2)	1.2	1.6 and 0.035	Cao et al. (2020)
BD-368-2	Single-cell sequencing (H)	RBD	Neutralize WT and PT SARS-CoV-2 infections	Direct ACE2-binding inhibition (compete with ACE2)	0.82	0.015 and 0.0012	Cao et al. (2020); Du et al. (2020)
BD-395	Single-cell sequencing (H)	RBD	Neutralize WT and PT SARS-CoV-2 infections	Direct ACE2-binding inhibition (compete with ACE2)	0.36	0.27 and 0.02	Cao et al. (2020)
BD-494	CDR3_H_ structure similarity (H)	S (RBD)	Neutralize PT (and WT) SARS-CoV-2 infections	Direct ACE2-binding inhibition (compete with ACE2)	0.69	0.024	Cao et al. (2020)
BD-503	CDR3_H_ structure similarity (H)	RBD	Neutralize PT (and WT) SARS-CoV-2 infections	Direct ACE2-binding inhibition (compete with ACE2)	0.24	0.016	Cao et al. (2020)
BD-504	CDR3_H_ structure similarity (H)	RBD	Neutralize PT (and WT) SARS-CoV-2 infections	Direct ACE2-binding inhibition (compete with ACE2)	0.32	0.033	Cao et al. (2020)
BD-505	CDR3_H_ structure similarity (H)	RBD	Neutralize PT (and WT) SARS-CoV-2 infections	Direct ACE2-binding inhibition (compete with ACE2)	1.2	0.012	Cao et al. (2020)
BD-507	CDR3_H_ structure similarity (H)	RBD	Neutralize PT (and WT) SARS-CoV-2 infections	Direct ACE2-binding inhibition (compete with ACE2)	1.3	0.07	Cao et al. (2020)
BD-508	CDR3_H_ structure similarity (H)	RBD	Neutralize PT (and WT) SARS-CoV-2 infections	Direct ACE2-binding inhibition (compete with ACE2)	1.9	0.015	Cao et al. (2020)
BD-515	CDR3_H_ structure similarity (H)	RBD	Neutralize PT (and WT) SARS-CoV-2 infections	Direct ACE2-binding inhibition (compete with ACE2)	0.041	0.022	Cao et al. (2020)
CA1; CB6	PBMC (H)	RBD, RBM (overlap ACE2-binding site)	Neutralize WT and PT SARS-CoV-2 infection	Direct hACE2-binding inhibition CB6 exhibits a steric hindrance	4.68;2.49	0.38; 0.036	Elshabrawy et al. (2012); Shi et al. (2020)
							
C121; C144; C135	PBMCs (H)	RBD, RBM, different from CR3022	Neutralize WT SARS-CoV-2	Direct ACE2-binding inhibition	NA	1.64E^−3^ 2.55E^−3^ 2.98E^−3^	Robbiani et al. (2020)
C1A-B3; C1A-F10; C1A-C2; C1A-H5; C1A-C4; C1A-B12; C1A-H6	PBMCs (H)	RBD, RBM, different from CR3022	Neutralize WT and PT SARS-CoV-2	Direct ACE2-binding inhibition	7.63 E^3^ 5.57 E^3^ 1.41 E^3^ 8.45 E^3^ 7.82 E^3^ 4.22 E^3^ 8.87 E^3^	0.008–0.671 0.062–441	Clark et al. (2020)
CC12.1–28	PBMC (H)	RBD, RBM (overlap ACE2-binding site)	Neutralize PT SARS-CoV-2 infections	Direct ACE2-binding inhibition	NA	0.01–1.00	Rogers et al. (2020)
CC6.16–19; CC6.32	PBMC (H)	RBD	Neutralize WT and PT SARS-CoV-2 infection	Direct ACE2-binding inhibition	NA	0.02–1.00	Rogers et al. (2020)
CC6.29–31	PBMC (H)	RBD, RBM (overlap ACE2-binding site)	Neutralize PT SARS-CoV-2 infections	Direct ACE2-binding inhibition	NA	0.001–0.002	Rogers et al. (2020)
CC6.33	PBMC (H)	RBD (distinct from ACE2-binding site)		Indirect ACE2-binding inhibition	NA	0.039	Rogers et al. (2020)
CM29	Ig-Seq proteomics (H)	S2	Low neutralization of WT SARS-CoV-2	Independent of hACE2-binding inhibition	6.6 nM	NA	Voss et al. (2020)
CM30; CM25; CM17; CM58	Ig-Seq proteomics (H)	NTD	Neutralize WT SARS-CoV-2	Independent of hACE2-binding inhibition	0.8 nM	0.83 0.01 0.03 0.81	Voss et al. (2020)
							
CM32	Ig-Seq proteomics (H)	RBD, ACE2	Neutralize WT SARS-CoV-2	Direct ACE2-binding inhibition	6.0 nM	2.1	Voss et al. (2020)
COV2-201_ (49 Abs)^b^	PBMC (H)	RBD and NTD	Neutralize WT SARS-CoV-2 infections	NA	NA	0.041–3.33	Zost et al. (2020b)
COV2-2130	PBMC (H)	S/RBD	Neutralize WT and PT SARS-CoV-2 infection	Independent of hACE2-binding inhibition	NA	0.12; 0.0016	Zost et al. (2020a)
COV2-2196	PBMC (H)	S/RBD, RBM (overlap ACE2-binding site)	Neutralize WT and PT SARS-CoV-2 infection	Direct ACE2-binding inhibition	NA	0.015; 0.007	Zost et al. (2020a)
COVA2-07; COVA2-39	PBMC (H)	RBD, RBM (overlap ACE2-binding site)	Neutralize WT and PT SARS-CoV-2 infection	Direct ACE2-binding inhibition	NA	0.001–0.1	Brouwer et al. (2020)
COVA1-18; COVA2-04; COVA2-15;	PBMC (H)	RBD (partially overlap ACE2-binding site)	Neutralize WT and PT SARS-CoV-2 infection	Direct ACE2-binding inhibition	NA	0.001–0.1	Brouwer et al. (2020)
							
COVA1-03; COVA1-21; COVA1-22	PBMC (H)	NTD	Neutralize WT and PT SARS-CoV-2 infection	hACE2-binding inhibition through steric hindrance	NA	NA	Brouwer et al. (2020)
							
CV07-209; CV07-250; CV07-270	PBMC (H)	RBD, RBM (overlap ACE2-binding site)	Neutralize WT SARS-CoV-2 infection	Direct ACE2-binding inhibition	NA	0.0031; 0.0035; 0.0823	Kreye et al. (2020)
							
CV1	PBMC (H)	RBD (distinct from ACE2-binding site)	Neutralize WT SARS-CoV-2 infection	Independent of hACE2-binding inhibition	NA	15	Seydoux et al. (2020)
CV30	PBMC (H)	RBD, RBM (overlap ACE2 binding site)	Neutralize WT SARS-CoV-2 infection	Direct ACE2-binding inhibition	3.6	0.03	Seydoux et al. (2020); Hurlburt et al. (2020)
DH1048; DH1049; DH1050-1; DH1050-2; DH1051	PBMC (H)	NTD	Neutralize WT and PT SARS-CoV-2	Similar to 4A8	16–19	0.039–0.52; 0.39–0.78	Li et al. (2021)
DH1042	PBMC (H)	RBD, RBM (overlap ACE2-binding site)	Neutralize WT and PT SARS-CoV-2	Direct ACE2-RBD inhibition (similar to H11-D4	NA	NA	Li et al. (2021)
DH1044	PBMC (H)	RBD, different from ACE2 binding site	Neutralize WT and PT SARS-CoV-2	Independent of hACE2-binding inhibition	NA	NA	Li et al. (2021)
EY6A	PBMC (H)	RBD (different from ACE2-binding site)	Neutralize WT SARS-CoV-2 infections	Independent of hACE2-binding inhibition	2.00	10.00	Zhou D. et al. (2020)
H2	PBMC (H)	RBD	Neutralize WT SARS-CoV-2 infection	Independent of hACE2-binding inhibition	14.3	1.00	Wu et al. (2020c)
H4	PBMC (H)	RBD (partially overlap B38-binding residues)	Neutralize WT SARS-CoV-2 infection, synergic effect with B38	Direct hACE2-binding inhibition	4.48	0.896	Wu et al. (2020c)
LY-CoV555; LY-CoV016	PBMC (H)	RBD (inside and outside RBM)	Neutralize WT and PT SARS-CoV-2 infection	Direct ACE2-binding inhibition	NA	NA	Jones et al. (2020); Chen et al. (2021)
P2B-1A1; P2B-1A10; P5A-1B6; P5A-1B8; P5A-1B9; P5A-2F11; P5A-2G7; P5A-2G9; P5A-3A1; P5A-3C12	PBMC (H)	S, RBD, same as CR3022 (369–386)	Neutralize WT and PT SARS-CoV-2	Direct inhibition of RBD-ACE2	0.75-90.09	0.0014–0.9231	Yan et al. (2020)
							
S2M11; S2E12	PBMC (H)	S/RBD (RBM from one S protein and RBD from adjacent S monomer)	Neutralize WT and PT SARS-CoV-2 infection	Direct ACE2-binding inhibition	66.0	3–6	Tortorici et al. (2020)
	PBMC (H)	S2E12 binds RBM (overlap ACE2-binding site)	Neutralize WT and PT SARS-CoV-2 infection	Direct ACE2-binding inhibition	1.6	1.9–2.5	Tortorici et al. (2020)
58G6; 510A5	PBMCs (H)	RBD, RBM (overlap ACE2 binding site)	Neutralize WT SARS-CoV-2	Direct ACE2-binding inhibition	0.385; 7.8	9.98; 11.13	Han et al. (2020)
XG012	PBMC (H)	S, S2	Neutralize WT and PT SARS-CoV-2	Inhibitition of Cell-virus fusion	NA	NA	Zhou et al. (2021)
XG006; XG027	PBMC (H)	S1, NTD	Neutralize WT and PT SARS-CoV-2	Steric hindrance	NA	NA	Zhou et al. (2021)
XG003-XG043	PBMC (H)	RBD	Neutralize WT and PT SARS-CoV-2	Direct inhibitition of RBD-ACE2	NA	6–15 ng/ml	Zhou et al. (2021)
REGN10989; REGN10987; REGN10933; REGN10934; REGN10977; REGN10964; REGN10954; REGN10984; REGN10986	PBMC (H/M)	RBD	Neutralize WT and PT SARS-CoV-2 infection	Direct ACE2-binding inhibition	3.7; 0.45; 3.4; 4.9; 3.0; 56.4; 2.4; 5.6; 0.7	7.2, 7.3; 0.4, 0.4; 0.4, 0.4 0.5, 0.3; 0.5, NA 0.6, NA 0.9, NA 1.0; NA 1.0, NA	Hansen et al. (2020)
Fab-298; Fab-52	Phage display	RBD	Neutralize WT and PT SARS-CoV-2	ACE2-binding inhibition	NA	NA	Rujas et al. (2020)
HTS0422; HTS0433; HTS0446; HTS0483	Phage display (H)	RBD	Neutralize WT and PT SARS-CoV-2 infection	Direct ACE2-binding inhibition	NA	0.179–0.231	Lou et al. (2020)
P17	Phage display (H)	RBD, RBM (overlap ACE2-binding site)	Neutralize WT and PT SARS-CoV-2 infection	Direct ACE2-binding inhibition	0.096	0.165; 0.195	Yao et al. (2021)
MD17; MD29	Phage display (H)	RBD, same as CR3022 (369–386)	Neutralize WT SARS-CoV-2	Steric hindrance	2.6; 0.4	43; 13	Noy-Porat et al. (2020)
MD45; MD65; MD67	Phage display (H)	RBD	Neutralize WT SARS-CoV-3	Direct inhibitition of RBD-ACE2	5.6; 2.5; 5.8	2.1; 0.22; 1.9	Noy-Porat et al. (2020)
MD62	Phage display (H)	RBD	Neutralize WT SARS-CoV-4	Direct inhibitition of RBD-ACE2	4.8	1.6	Noy-Porat et al. (2020)
MD47	Phage display (H)	RBD	Neutralize WT SARS-CoV-5	Direct inhibitition of RBD-ACE2	0.5	13	Noy-Porat et al. (2020)
n3086, n3113	Phage display (H)	S1/RBD (different from n3088/n3130 binding site and distinct from RBM)	Moderately neutralize PT SARS-CoV-2 infections	Independent of hACE2-binding inhibition	0.001; 0.002	26.6; 18.9	Wu et al. (2020a); Wu et al. (2020b)
n3088; n3130	Phage display (H)	S1/RBD (different from n3086/n3113 binding site and distinct from RBM)	Neutralize WT and PT SARS-CoV-2 infection	Independent of hACE2-binding inhibition	3 E^−5^; 1 E^−5^	3.3; 3.7	Wu et al. (2020a); Wu et al. (2020b)
S-B8; S-D4; S-E6	Phage display (H)	RBD, ACE2	Neutralize WT and PT SARS-CoV-2	Direct inhibitition of RBD-ACE2, and cell-virus fusion	170E^−3^; 10E^−3^; 110E^−3^	12.9; 7.1; 0.4	Qiang et al. (2020)
VH-Fc ab6; VH-Fc m397	Phage display (H)	RBD	Neutralize WT SARS-CoV-2 infections	Indirect ACE2-binding inhibition (compete with ACE2)	11 and 9.6	0.35 and 1.5	Sun et al. (2020)
1E2	Phage display (H)	RBD (overlap ACE2-binding site)	Neutralize WT and PT SARS-CoV-2 infection	Direct ACE2-binding inhibition	21.1	0.51, 0.31	Chi et al. (2020a)
2F2	Phage display (H)	RBD (partially overlap ACE2 epitope)	Neutralize WT and PT SARS-CoV-2 infection	Independent of hACE2-binding inhibition	0.84	0.411, 0.024	Chi et al. (2020a)
3F11	Phage display (H)	RBD (overlap ACE2 epitope)	Neutralize WT and PT SARS-CoV-2 infection	Direct ACE2-binding inhibition	3.15	0.436, 0.003	Chi et al. (2020a)
4D8	Phage display (H)	RBD,RBM (overlap ACE2 binding site)	Neutralize WT and PT SARS-CoV-2 infection	Direct ACE2-binding inhibition	33.97	0.455, 0.247	Chi et al. (2020a)
5F8	Phage display (H)	RBD (partially overlap ACE2-binding site)	Neutralize WT and PT SARS-CoV-2 infection	Independent of hACE2-binding inhibition	0.676	0.238, 0.003	Chi et al. (2020a)
Ty1-Fc; sdTy1	Phage display (Al)	S/RBD (distinct from ACE2-binding site)	Neutralize WT and PT SARS-CoV-2 infection	hACE2-binding inhibition through steric hindrance	9.0	0.77	Hanke et al. (2020)
NM1226; NM1228; NM1230	Phage display (Al)	RBD, slightly overlap ACE2 binding site	Neutralize WT and PT SARS-CoV-2	Steric hindrance	3.66; 1.37; 8.23	0.85; 0.5; 2.12 nM	Wagner et al. (2020)
							
aRBD-2; aRBD-3; aRBD-5; aRBD-7; aRBD-41; aRBD-54.	Phage display (Al)	RBD	Neutralize WT SARS-CoV-2	Direct inhibition of RBD-ACE2	2.60; 3.33; 16.3; 3.31; 21.9; 5.49	33–100	Ma et al. (2021)
							
aRBD-2-5; aRBD-2-7	Phage display (Al)	RBD	Neutralize WT SARS-CoV-2	Direct inhibition of RBD-ACE2	0.0592; 0.25	0.0118; 0.00676	Ma et al. (2021)
Nb11–59, Nb16–68	Phage display (Ca)	RBD (Nb11–59 can bind to S)	Neutralize WT and PT SARS-CoV-2 infection	Block SARS-CoV-2 RBD/ACE2 binding	0.21; 0.36	0.05; 2.20	Gai et al. (2020)
Nb4-43; Nb14–33; Nb15–61; Nb15–52; Nb16–75.	Phage display (Ca)	RBD (distinct from ACE2-binding site)	Neutralize WT and PT SARS-CoV-2 infection	Block SARS-CoV-2 RBD/ACE2 binding	0.48; 0.33; 0.10; 0.69; 0.31	NA	Gai et al. (2020)
Nanosota-1	Phage display (Ca)	RBD, RBM (overlap ACE2-binding site)	Neutralize WT and PT SARS-CoV-2	Direct inhibition of RBD-ACE2	0.0155	0.16	Ye G. et al. (2020)
Ig F (ab')_2_	Equine plasma (Eq)	RBD	Neutralize WT SARS-CoV-2 infection	Independent of hACE2-binding inhibition	0.76	0.07	Pan et al. (2020)
NIH-CoVnb-112	Phage display (Lla)	RBD	Neutralize WT SARS-CoV-2 infections	Direct ACE2-binding inhibition	5.00	0.02	Esparza and Brody (2020)
VHHs	PBMC-based library (Lla)	S	Neutralize SARS-CoV-2 infections	NA	0.25	1.0	Dong et al. (2020)
H11-H4 H11-D4	Phage display (Lla)	RBD	Neutralize WT SARS-CoV-2 infections	Direct ACE2-binding inhibition	5; 10	34; 28	Huo et al. (2020)
Nb89; Nb20; Nb21	Proteomic (Lla)	RBD, ACE2-binding site (for Nb20 & 21)	Neutralize WT and PT SARS-CoV-2	Direct inhibitition of RBD-ACE2	0.108; 0.0104 (for 89 and 20)	2.1E^−3^; 1.6E^−3^; 0.7E^−3^ and 2.4E^−3^; 0.8E^−3^; 0.4E^−3^	Xiang et al. (2020)
3A2; 3B3; 3C2; 3C6; 3G7; 3H2; 3H6	Hybridoma (M)	RBD, RBM (overlap ACE2-binding site)	Neutralize WT and PT SARS-CoV-2	Direct inhibition of RBD-ACE2	0.12 - 4.8	10 - 100	Chapman et al. (2020)
6D3	Hybridoma (M)	S; S1/S2 furin cleavage site	Neutralize WT SARS-CoV-2	Block access of host cell proteases, TMPRSS2 or furin, to the cleavage site	NA	0.16–0.8	Cheng et al. (2020)
1B10, 2B04, 1B07, 1E07	PBMC (M)	S, RBD	Neutralize WT and PT SARS-CoV-2	Direct ACE2-binding inhibition	NA	1.46 (for 2B04)	Alsoussi et al. (2020)
2H04	PBMC (M)	RBD, different from ACE2 binding site	Neutralize WT and PT SARS-CoV-2	Block the viral fusion to cell membrane	NA	NA	Alsoussi et al. (2020)
47D11	ELISA-cross reactivity (M)	RBD (S1B, residues 338–506)	Neutralize WT SARS-CoV-2 infection	Independent of hACE2-binding inhibition	9.6	0.061	Wang et al. (2020a); Wang et al. (2020b)
HB27, Fab-HB27	Phage display (M)	RBD, slightly overlap ACE2	Neutralize WT and PT SARS-CoV-2	Steric hindrance, conformational change that hampers the up conformation of S protein	0.27; 0.07	0.04 nM	Zhu L. et al. (2020)
H014 and its sd Fab	Phage display (M)	RBD (different from RBM)	Neutralize WT and PT SARS-CoV-2 infection	hACE2-binding inhibition through steric hindrance	0.096	0.53; 0.042	Lv Z. et al. (2020)
15031; 15032; 15033; 15034	Phage display (Sy)	RBD	Neutralize WT SARS-CoV-2 infections	Direct ACE2-binding inhibition	0.4; 0.8; 0.2; 0.8	0.57; 0.61; 0.185, 1.10	Miersch et al. (2020)
ACE2-Ig; mACE2-Ig	Genetic recombination (Sy)	RBD (overlap ACE2-binding site)	Neutralize PT SARS-CoV-2 infections	Indirect ACE2-binding inhibition	10	0.1 and 0.08	Lei et al. (2020)
C3; C7; C14; C17; C18; Co1; Co2; Co4	Machin learning (Sy)	NA	Neutralize SARS-CoV-2 infection	NA	NA	NA	Magar et al. (2020)
							
MR3; MR4; MR17; SR4	Ribosome and phage display (Sy)	RBD (overlap ACE2-binding site)	Neutralize PT SARS-CoV-2 infections	Direct ACE2-binding inhibition	1.00; 23.3; 83.7; 14.5	0.4; 0.74; 12.32; 5.9	Li T. et al. (2020)
910–30	Yeast display (H)	ACE2, a glycan region	Neutralize WT and PT SARS-CoV-2	Direct ACE2-binding inhibition, through S protein disassembly	0.230	0.071; 0.142	Banach et al. (2021)
NBP10, NBP11	Yeast display (H)	RBD, different from ACE2binding site	Neutralize WT SARS-CoV-2	Direct ACE2-binding inhibition	4.1; 0.18	13; 1.5	Bell et al. (2021)
ABP18	Yeast display (H)	RBD, RBM (overlap ACE2-binding site)	Neutralizes WT SARS-CoV-2	Direct ACE2-binding inhibition	1.8	0.06	Bell et al. (2021)
Nb11, Nb12; Nb15; Nb19	Yeast display (Sy)	S/RBD (overlap ACE2-binding site)	Neutralize PT SARS-CoV-2 infections	Direct ACE2-binding inhibition	76–800	16–335	Schoof et al. (2020)
Nb17	Yeast display (Sy)	S but not to RBD	Neutralize PT SARS-CoV-2 infections	hACE2-binding inhibition through steric hindrance	NA	106	Schoof et al. (2020)
Nb3	Yeast display (Sy)	S but not to RBD	Neutralize WT and PT SARS-CoV-2 infection	hACE2-binding inhibition through steric hindrance	NA	41.9; 54.5	Schoof et al. (2020)
Nb6	Yeast display (Sy)	S/RBD (overlap ACE2-binding site)	Neutralize WT and PT SARS-CoV-2 infection	Direct ACE2-binding inhibition	3.3 10^–6^	4.1 10^–6^	Schoof et al. (2020)
rRBD-15	Phage library (Sy)	RBD	Neutralize PT SARS-CoV-2 infection	Direct ACE2-binding inhibition	3.8	12.0	Zeng et al. (2020b); Zeng et al. (2020a)
RU (169–178; 167–230; 171–155)	Retained display (Sy)	RBD	Neutralize live SARS-CoV-2 infections	Direct ACE2-binding inhibition	1–400	0.88; 40.00; 30.00	Beasley et al. (2020)
RBD11i12; RBD1i1; RBD1il3; RBD6id	Yeast display (AHEAD)	RBD, RBM (overlap ACE2-binding site, for Nb20 & 21)	Neutralize WT and PT SARS-CoV-2	Direct inhibition of RBD-ACE2	316; 48.1; 32.2; 263	0.04; 0.18; 0.05; 0.056	Wellner et al. (2020)
RBD10i10; RBD10i14; RBD3i17; RBD3i2	Yeast display (AHEAD)	RBD	Neutralize WT and PT SARS-CoV-2	Direct inhibition of RBD-ACE2	2.14; 0.72; 230; 128	0.19; 0.42; 0.116; 5.52	Wellner et al. (2020)
Sb23		RBD (distinct from ACE2 epitope)	Neutralize PT SARS-CoV-2 infections	hACE2-binding inhibition through steric hindrance	10.6	0.6	
IgG1 ab1	Phage display	S/RBD	Neutralize WT and PT SARS-CoV-2 infection	Direct ACE2-binding inhibition	0.16	0.01	Li W. et al. (2020)
4A3	Phage display	RBD	Neutralize PT SARS-CoV-2 infection	Indirect ACE2-binding inhibition	3.2	0.28	Liu X. et al. (2020)
4A10; 4A12; 4D5	Phage display	RBD	Neutralize WT and PT SARS-CoV-2 infection	Indirect ACE2-binding inhibition	1.03; 2.47; 5.82	0.66; 0.19; 1.13	Liu X. et al. (2020)

H, Human; Hz, Humanized; Al, Alpaca; Ca, Camel; Eq, Equine; Lla, Llama; M, Mouse; Sy, Synthetic; WT, wild-type (or authentic) SARS-CoV-2; PT, pseudotyped SARS-CoV-2. K_D,_ dissociation constant (in nM), IC_50_, half-maximal inhibitory concentration (in µg/mL but only in nM for rRBD-15, and pM for REGN10989, REGN10987, REGN10933, REGN10934, REGN10977, cREGN10964, REGN10954, REGN10984, REGN10986, and P17). NA, Non-attributed. ^a^This set of NmAbs contains 206 reported antibodies, among which the most potent are P2C-1F11, P2B-2F6, and P2C-1A3. ^b^This set of NmAbs contains 49 reported antibodies. For sets of NmAbs that neutralize both pseudovirus and authentic virus, if only one value of K_D_/IC_50_ is reported, this value is related to the authentic virus binding neutralization.

Firstly, among these human neutralizing antibodies (hNmAbs), hundreds of the reported demonstrated a specific activity only against SARS-CoV-2, but not against other Sarbecovirus (or coronavirus), and recognize different sub-domains localized only on SARS-CoV-2 S RBD. Specifically, almost all of these hNmAbs could neutralize both WT and PT viruses by only targeting RBD with significant affinities (*K*_*D*_ < 10 nM). Note that some but not all of the herein reported hNmAbs were tested for cross-activity against other coronaviruses. Although almost all of the reported hNmAbs exhibited a neutralizing effect with IC_50_ values less than 10 ng/ml against both WT and PT SARS-CoV-2, the main attention (but not limited) goes toward the 9 NmAbs isolated by Hansen *et al.* (Hansen et al., 2020) as well as BD-368-2, 5A6, 1-20, 1-57, 2-4, 2-7, 2-15, 2-36, 4-20, COV2-2130, COV2-2196, P17, ABP18, NBP10, NBP11, Nanosota-1, and S2E12 and S2M11 (Cao et al., 2020; Du et al., 2020; Liu L. et al., 2020; Tortorici et al., 2020; Wang B. et al., 2020; Ye G. et al., 2020; Zost et al., 2020a; Bell et al., 2021; Yao et al., 2021), as they are the most potent antibodies well described with high binding affinity. Noticeably, all these hNmAbs, but not COV-2310, target epitopes overlapping with the ACE2 binding site, known as the receptor-binding motif (RBM). BD-368-2, LY-CoV555, LY-CoV016 and the bivalent mutation-insensible hNmAbs 5A6 and S2M11 can entirely block ACE2 binding by burying two adjacent or all three RBDs simultaneously and according to the S state (either in their “up (or open)” or “down (or close),” or in both positions) and exhibit strong therapeutic and prophylactic effects against the WT and the PT virus (Cao et al., 2020; Jones et al., 2020; Tortorici et al., 2020; Wang B. et al., 2020). It has been shown that NBP10, NBP11, COVA2-04, CR3022, and EY6A share overlapping but distinct epitopes. However, unlike CR3022 which react, but does not neutralize SARS-CoV-2, NBP10 and NBP11 displayed abilities to block and protect host cells against SARS-CoV-2 (Bell et al., 2021), making NBP10 and NBP11 of a great importance as they reach CR3022 epitope that is cryptic.

Unlike other RBD-specific hNmAbs presented in Table 3 as burying the same epitope with ACE2 and displaying direct prevention of ACE2 to bind RBD, BD-368–2, 5A6, REGN10989, REGN10987, REGN10933, REGN10934, and S2M11 neutralize SARS-CoV-2 through an additional mechanism that is a steric hindrance, which enhances the disruption effect of each of the hNmAbs for the ACE2-binding formation. COV2-2130 and COV2-2196, on the other side, exhibit an enhanced synergic neutralization and protection against WT and PT SARS-CoV-2 infection through respective independent neutralization mechanisms, as COV2-2196 targets the overlapping ACE2-binding site while COV2-2130 target a different epitope on RBD (Hansen et al., 2020; Zost et al., 2020a). S2M11, by binding to adjacent RBDs, keeps the virus in "down" conformation, inhibiting its binding to ACE2.

Fifteen hNmAbs namely 58G6; 510A5, B5, H2, B38, EY6A, CV1, CV30, CV07-209, H4, 5C2, 1G6, VH-Fc ab6, VH-Fc m397, and 1C10 exhibit potential neutralization and protection activities only against WT SARS-CoV-2 infection, but not against the PT virus, by targeting epitopes on RBD (Chi et al., 2020b; Han et al., 2020; Hurlburt et al., 2020; Kreye et al., 2020; Seydoux et al., 2020; Sun et al., 2020; Wu et al., 2020c; Zhou D. et al., 2020). Apart from B5, H2, EY6A, CV1, and 1C10, which hamper the WT SARS-CoV-2 S protein to bind ACE2 by an indirect inhibition mechanism, the seven hNmAbs remaining exhibit a direct blocking of ACE2-RBD formation, as these later recognize epitopes overlapping ACE2 binding site. B5, with the lowest affinity to RBD (*K*_*D*_ *=* 305 nM), exhibits a partial competition with ACE2 to bind RBD, therefore suggested to be a weak neutralizer (Wu et al., 2020c).

Secondly, the few hNmAbs that could only neutralize both WT and PS SARS-CoV-2 target, in addition to RBD, others epitopes on SARS-CoV-2 S protein, among which, the N-terminal domain (NTD). Chi et al. (Chi et al., 2020b) and Liu et al. (Liu L. et al., 2020) reported the isolation of 21 (2 and 19 respectively) potent therapeutic hNmAbs, which exhibited a strong neutralization activity against WT SARS-CoV-2 and PT SARS-CoV-2. Among them, nine (1-20, 1-57, 2-4, 2-7, 2-15, 2-30, 2-36, 2-38 and 4-20) target RBD with little or no binding to NTD, nine (4A8, 1-68; 1-87, 2-17, 4-8, 4-18, 4-19, 4-32 and 5-24) target NTD and two (2-43 and 2-51) target S epitopes by burying epitopes on different domains. The first set of the nine hNmAbs could display an inhibition depending on a direct restriction also with a steric hindrance of ACE2 to link with SARS-CoV-2 RBD, as they strongly compete with ACE2 and some of them bind to NTD. Antibodies 2-43 and 2-51, together with XG006 and XG027 appeared to target epitopes obtained in the quaternary structure of S bridging RBD and NTD, and with the second set of NmAbs, 2-43, 2-51, XG006 and XG027 exhibit a steric hindrance-based inhibition. By neutralizing SARS-CoV-2 through NTD and/or RBD binding (Liu L. et al., 2020; Zhou et al., 2021), 2-43, 2-51, XG006, and XG027 are good therapeutic targets. An *in silico* screening assay from 48A revealed that aside from binding to NTD, 4A8 binds to S1/S2 furin cleavage site (Cheng et al., 2020), hampering the furin protease to access the site, which consequently inhibits a step toward the viral entry. Similar to 4A8, antibodies DH1048, DH1049, DH1050-1, DH1050-2, and DH1051 demonstrated the same binding characteristics, neutralizing wild-type and pseudotype SARS-CoV-2 (Li et al., 2021).

The only five humanized single domain antibodies (nanobodies), 1E2, 2F2, 3F11, 4D8, and 5F8, obtained from phage display neutralize WT and PT SARS-CoV-2 by targeting residues on ACE2 binding site and prevent against both virus infection types through a direct blocking of RBD-ACE2 binding. This activity was considerably enhanced when fused to Fc as complete IgG antibodies. 1E2, 3F11, and 4D8 completely prevented SARS-CoV-2 RBD-ACE2 binding, while 2F2 and 5F8 partially prevent this binding. None of them, except for 5F8, bound to SARS-CoV (Chi et al., 2020a).

Besides the S1-specific hNmAbs, only five hNmAbs (0304-3H3, 6D3, 9A1, CM29, and XG012) showing to be specific to the SARS-CoV-2 S2 subunit have been reported (Cheng et al., 2020; Chi et al., 2020b; Voss et al., 2020; Zhou et al., 2021). 0304-3H3, exhibited the highest neutralizing capacity (IC_50_: 0.04 μg/ml) against the WT, but not the PT SARS-CoV-2, while 9A1 and CM29 exhibited very weak protection against SDARS-CoV-2 (Chi et al., 2020b; Voss et al., 2020). 6D3 inhibits infection by blocking the access of host cell proteases, TMPRSS2 (or furin protease), to the cleavage site.

### Non-Human SARS-CoV-2 NmAbs

Numerous technologies have been used and improved to develop potent neutralizing antibodies with high protection efficacy against SARS-CoV-2, with the phage surface display representing the most utilized production technology. Of all the NmAbs from non-human sources, only one group of developed nanobodies (Nb3 and Nb17) obtained through synthesis exhibit robust protection against PS SARS-CoV-2 infection by targeting an epitope on S, away from RBD (Schoof et al., 2020). Therefore, this group of synthetic antibodies (sybodies) prevent the virus from entering the target cells through an allosteric inhibition mechanism. The remaining nhNmAbs against SARS-CoV-2 recognize RBD **(** Table 3).

Specifically, 20 of these reported SARS-CoV-2 RBD-specific nhNmAbs including ACE2-Ig, mACE2-Ig (Lei et al., 2020), MR3, MR4, MR17, SR4 (Li T. et al., 2020), rRBD-15 (Zeng et al., 2020a; Zeng et al., 2020b), IgG1 ab1 (Li W. et al., 2020), 15031, 15032, 15033, 15034 (Miersch et al., 2020), RU169–178, RU167–230, RU171–155 (Beasley et al., 2020), Nb6 (Schoof et al., 2020), and Nb11, Nb12, Nb15, Nb19 (Schoof et al., 2020), were demonstrated to target RBD-epitopes overlapping with ACE2 binding site directly. Besides, recently, Ma et al. (Ma et al., 2021) developed six efficient alpaca RBD-specific nanobodies, including aRBD-2, aRBD-3, aRBD-5, aRBD-7, aRBD-41, and aRBD-54, and more interestingly, engineered two hetero-bivalent nanobodies named aRBD-2-5 (from aRBD-2 and aRBD-5) and aRBD-2-7 (from aRBD-2 and aRBD-7) with higher potent neutralizing ability against WT SARS-CoV-2 (Ma et al., 2021). Thus, by competing with ACE2 in ELISA assays, these antibodies exhibit a direct ACE2-binding inhibition, while the remaining SARS-CoV-2 RBD-specific NmAbs display a different neutralization mechanism, suggested to be a steric hindrance.

### SARS-CoV-2 NmAbs with Cross-Activity

Among the NmAbs tested for their cross-activity against other coronaviruses, XG014, 5F8, CC6.33, ACE2-Ig, mACE2-Ig, H014, and its related single domain antibody H014-Fab, EY6A, and aRBD-41 have been reported as showing a cross-binding to SARS-CoV, and all, but not 5F8 and aRBD-41 could potently cross-neutralize SARS-CoV (Chi et al., 2020a; Lei et al., 2020; Rogers et al., 2020; Zhou D. et al., 2020; Ma et al., 2021; Zhou et al., 2021). Interestingly, these NmAbs were demonstrated to recognize conserved residues between the RBDs of SARS-CoV and SARS-CoV-2. None of the tested SARS-CoV-2-specific NmAbs was found to cross-react with MERS-CoV.

## Antigenic Epitope and Molecular Action Mechanism

**FIGURE 1:**
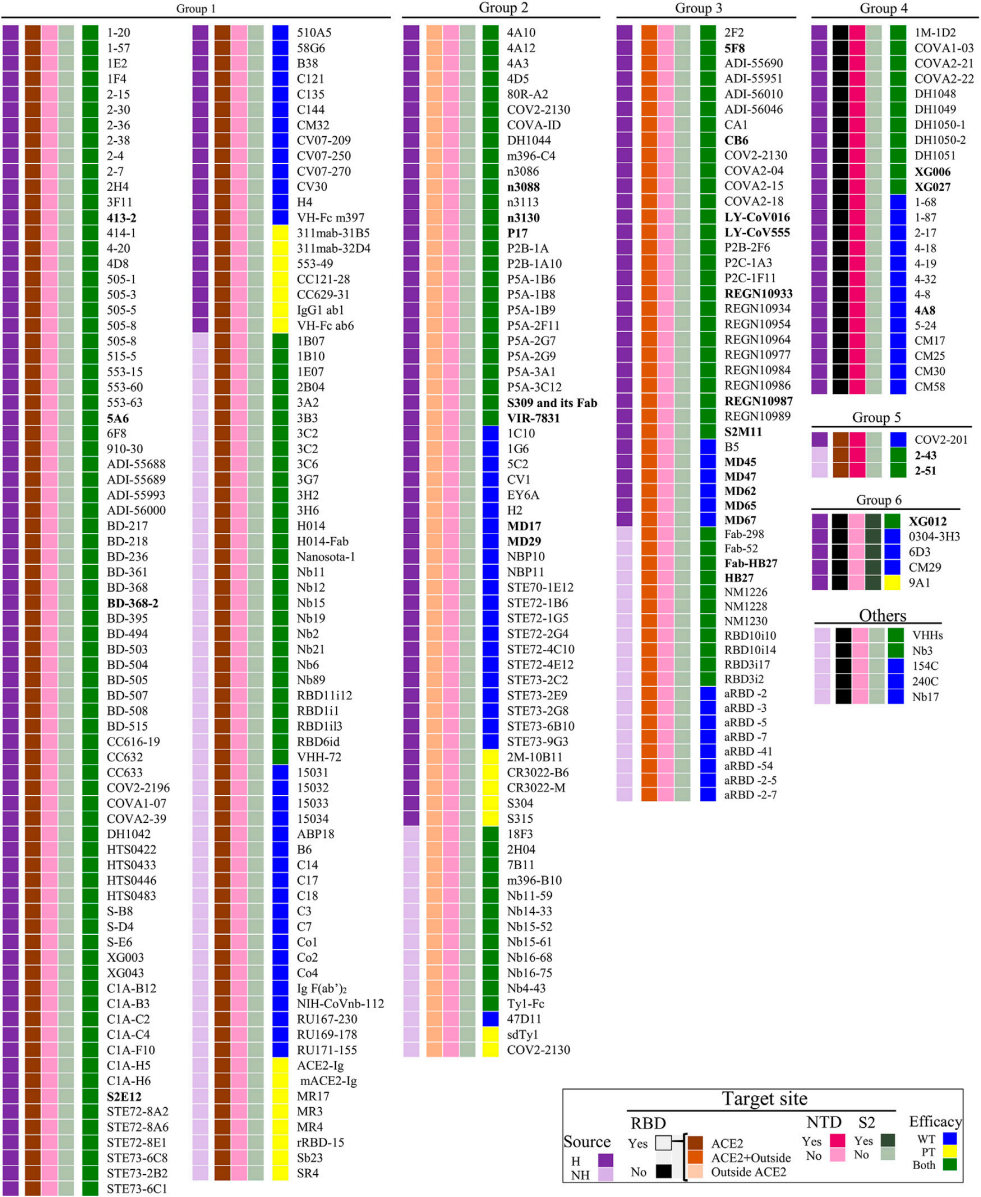
Potential target site-based SARS-CoV-2 NmAbs classification. H, Human NmAbs; NH, non-human NmAbs; ACE2, angiotensin-converting enzyme 2; NTD, N-terminal domain; WT, wild-type SARS-CoV-2; PT, pseudotyped SARS-CoV-2 (right squares). NmAbs in bold font are the potential antibodies we suggest in Figure 4 to consider in the cocktail formulation. The functions of represented NmAbs groups are described hereinbefore and illustrated in Figure 2C.

**FIGURE 2:**
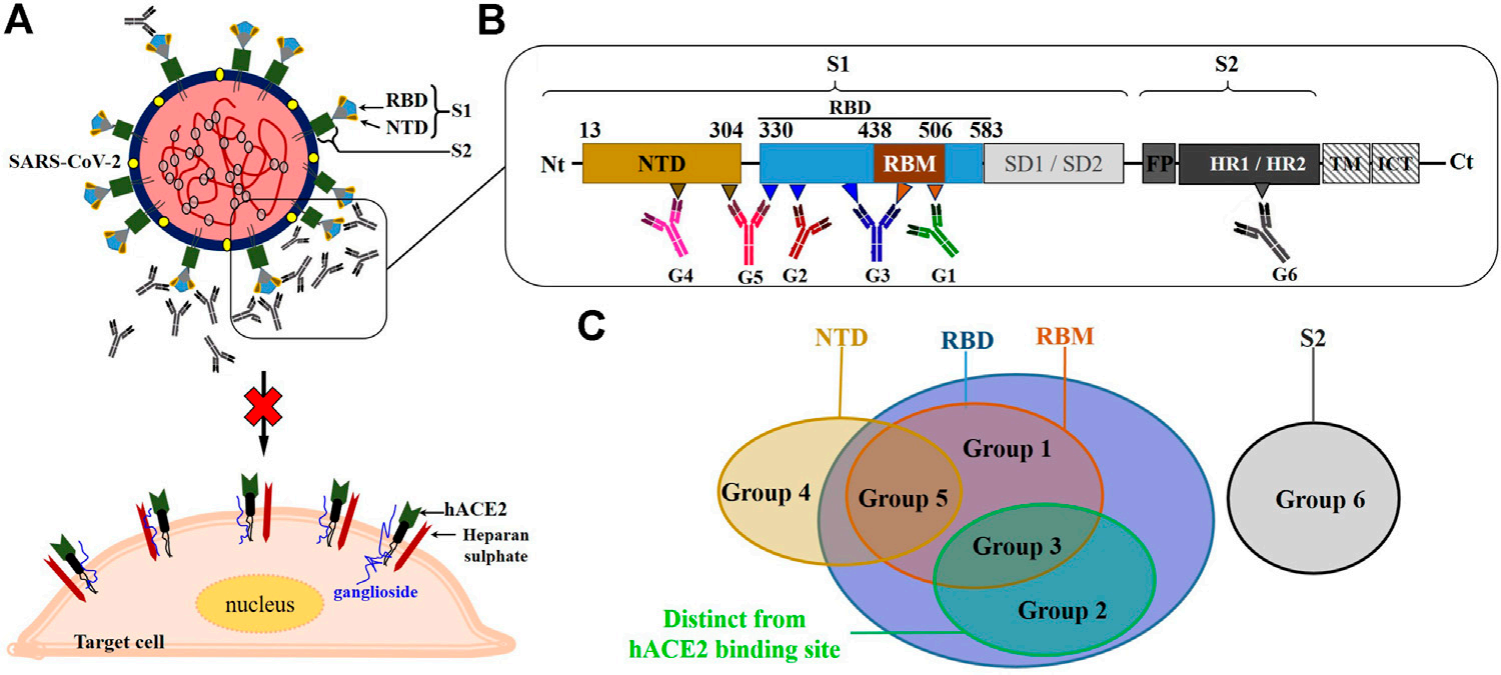
Overview of the schematic representation of the neutralization mechanism of NmAbs against SARS-CoV-2. **(A)**: NmAbs block SARS-CoV by targeting surface viral spike protein. **(B)**: Schematic representation of the monomeric SARS-CoV-2 spike protein and the neutralizing antibody binding fashions at their respective targeted regions **(C)**. NTD, N-terminal domain; RBD, receptor-binding domain; RBM, receptor binding-motif; SD1, subdomain 1; SD2, subdomain 2; FP, fusion peptide; HR1, heptad repeat 1; HR2, heptad repeat 2; TM, transmembrane domain; ICT, intracellular tail.

**FIGURE 3:**
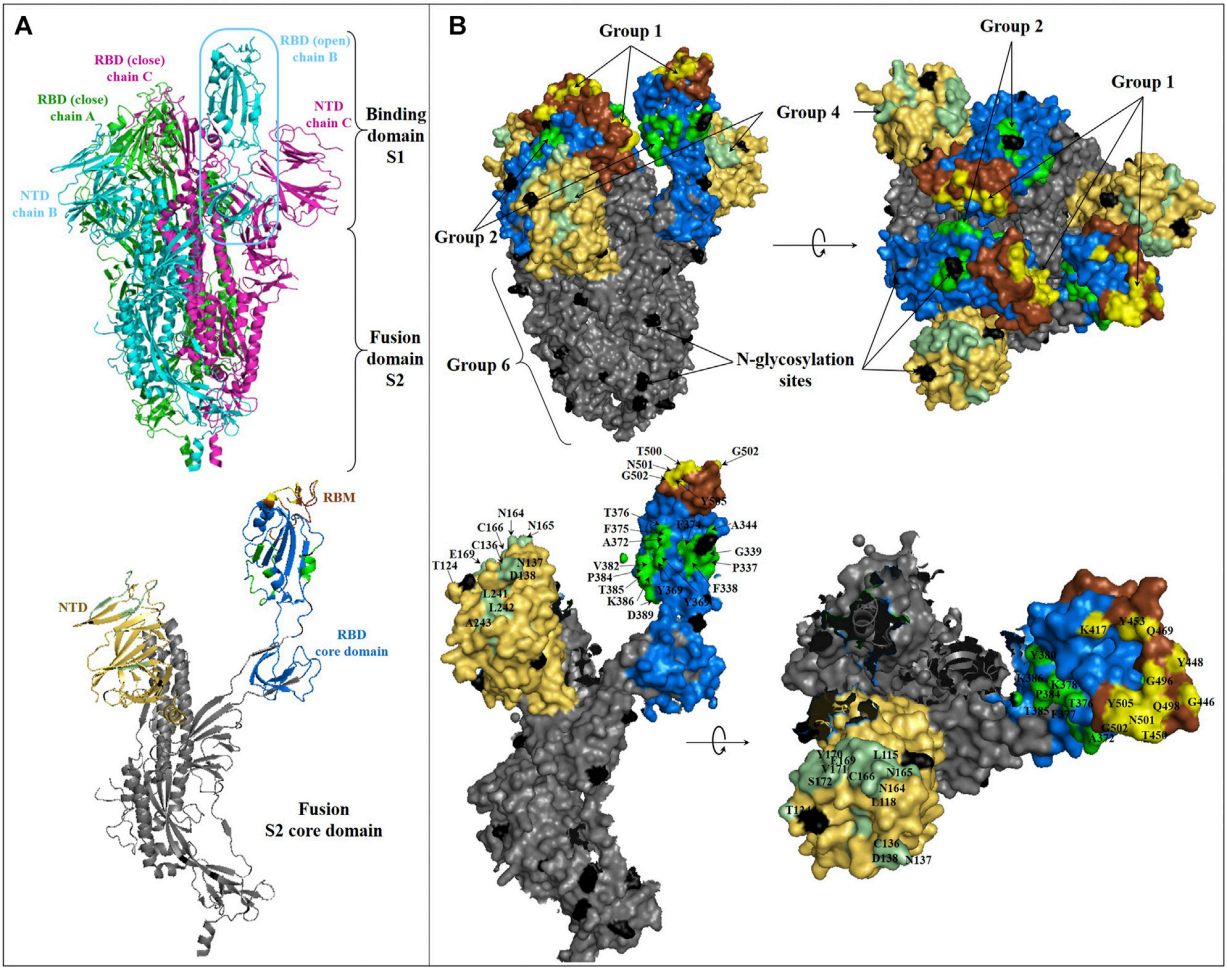
Structure of SARS-CoV-2 spike glycoprotein and representation of the main antibody-targeted regions. **(A)**: Overall crystal structure in cartoon representation of trimeric **(top)** and monomeric **(bottom)** SARS-CoV-2 Spike protein with one chain B in the open conformation (PDB:6VYB). The NTD, RBD, RBM, and S2 domains in monomeric representation **(bottom)** are colored as in Figure 2B. **(B)**: Binding surface/interface of the described NmAb groups on SARS-CoV-2 S glycoprotein. The RBD core domain is colored in blue, as in Figure 2B. The competitive binding surface of hACE2 with most of the described RBM-specific NmAbs, are colored in yellow [hACE2 binging epitope comprising K417, G446, Y449, Y453, L455, F456, A475, F486, N487, Y489, Q493, G496, Q498, T500, N501, G502, and Y505 (Lan et al., 2020)]. The epitope surface of NmAbs on RBD outside of RBM is colored in green (based on S309 (Pinto et al., 2020a), EY6A (Zhou D. et al., 2020), and CR3022 (Yuan M. et al., 2020) binding epitopes). Residues of surface S1 outside of RBD are not represented but are located within the RBD core domain colored in blue. The S2 binding surface, including HR1 and HR2, is colored in dark grey. The NTD-binding interface is colored in pale green (based on 4A8, with residues Y145, H146, K147, K150, W152, and R246 missing in this structure). N-glycosylation sites are colored in black. Residues involved in some main SARS-CoV-2 S and NmAbs interaction surfaces are labeled.

From the previous general description of the previously mentioned NmAbs and their binding characterizations, we note several antigenic neutralization sites within the SARS-CoV-2 S protein, with most of them localized onto the S1 subunit. Only few NmAbs have been reported against the SARS-CoV-2 S2 subunit so far, while numerous S2-specific NmAbs are reported for SARS-CoV and MERS-CoV (Elshabrawy et al., 2012; Wang et al., 2015). Thus, all the NmAbs reported here can be classified into six groups (Arabi et al., 2016; Jean et al., 2020; Li C. et al., 2020; Wu F. et al., 2020; Yang et al., 2020; Zhu N. et al., 2020), consisting of 3 sets (I-III) distributed over S1 (RBD and NTD) and S2, according to the S protein neutralization target site and the neutralization fashion (Figures 1–3).

The first set (set I) of NmAbs consists of antibody groups 1, 2, and 3, targeting RBD's epitopes. Specifically, group 1 contains NmAbs targeting only RBM, i.e., overlapping with ACE2-binding site (named as RBM-specific NmAbs). Group 2 includes antibodies that target RBD epitopes away from the ACE2-binding site so that they do not overlap ACE2 binding site (like the CR3022-binding site). Group 3 contains NmAbs, which bury large areas in RBD, including both the ACE2-binding site and other RBD sites. The NmAbs set II only includes the group 4 targeting epitopes localized on NTD. The only two NmAbs (2-43 and 2-51) (Liu L. et al., 2020), which constitute group 5, are contained in both sets II and III, as these neutralizing antibodies block the SARS-CoV-2 virus by binding to discontinuous epitopes localized onto different domains of the trimeric spike protein, notably NTD and RBD. The third set (set III) contains group 6, antibodies that target the S2 spike protein sub-unit. 0304-3H3 and 9A1, which strongly neutralize WT and weakly neutralize PT SARS-CoV-2, respectively, are the only NmAbs reported so far for this group 6.

Since the reported NmAbs target multiple therapeutic sites on S protein, different action mechanisms are associated with each NmAb group. As a summary of what has been already described hereinbefore, the group 1 NmAbs neutralizes SARS-CoV-2 through a direct RBD-ACE2-binding inhibition.

The group 2 NmAbs that target RBD outside of the receptor-binding motif (RBM) hamper SARS-CoV-2 RBD to bind ACE2 through a steric hindrance by burying close and very close residues to RBM (Custódio et al., 2020; Tai et al., 2020b). Moreover, the binding of some NmAbs of the group 2 at their neutralization sites, like EY6A, for instance, leads to spike protein conformation changes, which is hypothetically responsible for either a significant modification of the ACE2-binding site conformation or a steric occupancy of the ACE2-binding site by the bonded antibody, therefore preventing the S/RBD-ACE2 complex formation (Zhou D. et al., 2020). Besides that, it is worthy to note that the binding to RBD epitope away from that of the ACE2-binding site has been revealed to lead to the blockage of RBD to adopt the “open” conformation, supposedly required for ACE2-binding, thus preventing the viral entry into cells. This mechanism was observed for S2M11 (Tortorici et al., 2020), a group 3-specific NmAb, which partially overlaps the group 2 binding site.

Thus, group 3 blocks SARS-CoV-2 through combined mechanisms described for group 1 and group 2 NmAbs. Interestingly, a recent report found that HSs on the cell surface are involved in the binding to SARS-CoV-2 RBD (Clausen et al., 2020). A group of positively charged amino acid residues located within SARS-CoV-2 RBD, away from RBM, first mediates the binding to cellular HS, promoting the RBD to move from the “close” to the “open” conformation, suitable for ACE2 binding. As almost all of the positively charged amino acids are located at the N-terminal region of RBD, this finding has revealed an important role played by this region in the infection process and helps to understand the possible related mechanism of antibody neutralization. Therefore, the NmAbs from groups 2 and 3, targeting totally or partially this region, directly hamper this first binding and indirectly prevent subsequent host-virus interactions. This finding is consistent with the mechanism of action of S2M11 (Tortorici et al., 2020) described above, which prevents the trimeric spike protein from adopting the “open” conformation.

The role(s) played by SARS-CoV-2 NTD in the membrane fusion and the cell entry during the infection cycle is still not fully well defined. It has been recently reported that before the receptor binding interactions, a binding of SARS-CoV-2 NTD-specific residues (D111-S162), named as ganglioside binding domain (GBD) occurs, to cell membrane lipid rafts (Fantini et al., 2021). However, the binding at the recognition virus site of SARS-CoV-2 NTD-specific NmAbs (specifically 4A8), defined as group 4 antibodies, hampers the SARS-CoV-2 spike protein to bind onto cell surface receptors through a direct NTD-GBD-binding inhibition, subsequently resulting in non-formation of S-ACE2 complex. On the other hand, it has been previously demonstrated that the deletion of the SARS-CoV NTD residues (17-276), within which non-overlapped dimerization domains are located, completely abolishes the membrane fusion mediated by SARS-CoV S2 protein while retaining the receptor-binding function. Moreover, the SARS-CoV S lacking NTD leads to the monomeric state, which weakly binds cell surface receptors compared to the dimeric state, which exhibits a higher binding affinity to cell receptors. Thus, these suggest that the SARS-CoV NTD is required for the membrane fusion through a mechanism involving spike protein dimerization that is important for high affinity to cell receptors (Xiao et al., 2004). Therefore, as SARS-CoV-2 and SARS-CoV share similarities and exhibit similar infection mechanisms (Ceccarelli et al., 2020; Tai et al., 2020a; Tian et al., 2020; Wan Y. et al., 2020), and in comparison with what has been already demonstrated for the latter (SARS-CoV), we hypothesize that SARS-CoV-2 NTD, targeted by group 4 NmAbs might also be involved in the post-receptor binding steps of the cell entry process. Thus, once bound to the RBD upstream domain, the NTD-specific NmAbs would decrease the S protein affinity to cell receptors and hamper the post-receptor-binding steps necessary for SARS-CoV-2 to enter the target cells.

2-43 and 2-51 from group 5 block SARS-CoV-2 from entering cells through a mechanism of action coupling NTD- and RBD-related neutralization mechanisms, while 0304-3H3 and 9A1 from group 6 prevent the fusion of SARS-CoV-2 S with the cell membrane.

## Advantages of anti-SARS-CoV-2 NmAbs

### Complementary Biological Diagnostic Test

The use of isolated NmAbs would be of benefit in the serological diagnosis and monitoring of COVID-19 patients. Indeed, since the COVID-19 epidemic started, its diagnosis was mainly based on nucleic acid assessment through reverse transcription real-time PCR (RT-qPCR) from nasal and oropharyngeal samples. This gold standard—which in principle does not seem to be one (Dramé et al., 2020)—encountered a potential limit in COVID-19 monitoring, which is the existence of a non-negligible rate of aberrant results (false negatives) (Li D. et al., 2020). Falsely diagnosed negative patients were discharged from hospitals (Li Y. et al., 2020), which led to an increase in human-to-human contamination. However, the combination of RT-qPCR with the detection of serum antibodies made it possible to reduce the rate of false-negatives and quickly isolate truly positive patients from negative or naive people for better management and appropriate follow-up (Li Y. et al., 2020; Li Z. et al., 2020). To this, it might be added that the viral antigen detection through specific antibodies against the viral protein S would be supportive, as suitable to be used in indirect and sandwich ELISA (Zheng et al., 2020). Practically, these last two complementary serological diagnostic tests might be set up either as either an indirect or a direct diagnosis test (Li Y. et al., 2020) because they specifically target circulating antibodies and the viral protein, respectively. Besides, isotopic specific antibodies IgA, IgG, and IgM constitute basic biological markers for the follow-up of COVID-19 patients (Li Y. et al., 2020; Li Z. et al., 2020; Kontou et al., 2020), which can be associated with the search for the viral genome, even if the viral mutations due to immune pressure could compromise these specific ELISA tests.

### Possible Improvement of NmAbs Effects

As NmAbs can be highly effective in critically ill COVID-19 patients, they in immunotherapy can be efficiently improved as well, leading to a supportive potential reduction in viral load. Through engineer designing-dependent mutations to eliminate beneficial Fc-mediated effects such as antibody-dependent cell-mediated cytotoxicity (ADCC), appearing to have less or no effect in diminishing COVID-19 virus load, we possibly gain in terms of improvement of an effective SARS-CoV-2 neutralization, specifically during the acute phase of the illness (Klasse and Moore, 2020).

### Avoid ADE Effect and Infection Risks

The CPs have been shown to neutralize SARS-CoV-2, improve very severe cases, and reduce healing time (Ahn et al., 2020; Pawar et al., 2020; Rojas et al., 2020; Shen et al., 2020; Ye M. et al., 2020). Likewise, the existence of cross-reactivity or cross-neutralizing effect of plasmas obtained from SARS patients and mice has been reported against SARS-CoV-2 (Lv H. et al., 2020). Therefore, these therapeutic neutralizing effects are attributed to either specific- or cross-pAbs, containing a mixture of both neutralizing and non-neutralizing antibodies. However, it has been reported that the risk of developing ADE-related infections is associated with non-neutralizing antibodies taking precedence over neutralizing antibodies (Quinlan et al., 2020). By thus, producing NmAbs, the SARS-CoV-2 neutralization efficiency is highly enhanced, and the risk of developing ADE-related infections might be avoided, as the products are pure and specific. Therefore, the efficacy of isolated NmAbs in therapy would be maximized and better when used in high concentration because it has been observed in SARS-CoV infection that highly diluted or less concentrated NmAbs lead to antibody-dependent SARS-CoV infection severity (Wang et al., 2014; Negro, 2020; Quinlan et al., 2020).

On the other hand, although improved blood banking technics reduce transfer blood-borne pathogens-related risks/diseases, the use of CP in prophylaxis and therapy is noteworthy to be associated with particular diseases (Bloch et al., 2020; Casadevall and Pirofski, 2020). For instance, as *Casadevall and Pirofski* reviewed (Casadevall and Pirofski, 2020), the main receivers of CP in the context of COVID-19 would likely be people with pulmonary pathology or breathe discomforts, in whom risks for developing transfusion-related acute lung injury (TRALI) after plasma administration are non-negligible. Therefore, the use of specific and highly pure NmAbs as immunotherapeutic would highly exclude such pathology risks.

### Conserved Sarbecovirus Blocking Sites

It is surprising that after the emergence of the two previous SARS and MERS coronavirus epidemics and the inter-human circulation of other HCoVs, the extensive researches launched to eradicate these diseases over the past ten years could still not develop vaccines or drugs against coronavirus, especially against sarbecovirus family viruses. Not even a well-proven and conclusive treatment against coronavirus-related diseases, based on supportive and randomized significant studies has been adopted. By reviewing the epidemiology of the SARS- and MERS-CoV-related events, it seems that, because these epidemics have been under control, it has discouraged researchers from developing anti-coronavirus therapeutics. sarbecoviruses are a subgenus of β-coronaviruses that contain SARS-CoV, SARS-CoV-2, and other circulating HCoVs. Studies on the isolation of NmAbs against SARS-CoV-2 have revealed the existence of conserved neutralization sites in sarbecoviruses capable of being reached by cross-neutralizing antibodies (Bertoglio et al., 2020; Wec et al., 2020). This is very beneficial as it suggests that the use of broad sarbecovirus neutralizing antibodies as an immunotherapeutic cocktail would be an option to help mitigate not only COVID-19 but also other HCoVs associated diseases, as well as preventing the emergence of other sarbecovirus mutants. However, concerns still hang on the fact that the broad-spectrum neutralizing antibodies seem less effective—in terms of neutralization effect and escape mutants blocking potency—and more likely to lead to ADE effects than the specifics neutralizing antibodies (Cao et al., 2020; Pinto et al., 2020b; Robbiani et al., 2020; Zost et al., 2020a).

### Divers NmAbs for Several Blocking Sites

One of the most important advantages in developing NmAbs to treat the COVID-19 pandemic is the presence of several neutralization sites within the most immunogenic protein, Spike protein (Figures 2, 3). Firstly, based on the antibody-binding sites, three (Arabi et al., 2016) main regions are classified as target sites for SARS-CoV-2 neutralization (RBD, NTD, and S2). Within RBD, NTD and S, 3, 1, and 1 attack sites are respectively defined (Figures 1–3). Administration of cocktails consisting of a combination of NmAbs targeting different and non-overlapping sites, particularly within RBD (where lie selective amino acid residues for escape immunity) could favorably and effectively block infection during the early stages of the cell cycle (Pinto et al., 2020a) and limit the transmission. Moreover, these NmAbs cocktails, which can block WT and PT SARS-CoV-2, would neutralize and prevent possible escape variants that could arise from immune pressure (Baum et al., 2020b).

### Antibody Cocktails and Treatments

While the US Food and Drug Administration (FDA) has approved convalescent plasmas (CPs) as a treatment in critically ill COVID-19 patients (Tanne, 2020), based on the proven cure effect of CPs without significant reported adverse effects (Ahn et al., 2020; Pawar et al., 2020; Rojas et al., 2020; Shen et al., 2020; Tanne, 2020; Ye M. et al., 2020), there are still lots of limitations in using CPs. These limitations include i) the risks associated with collecting samples and obtaining large-scale plasmas to treat the world wild infected population, ii) the risks associated with ADE effects (Bloch et al., 2020), and particularly iii) the lack of strong reliable controlled and randomized studies supporting the real therapeutic effect of CPs, as several patients received antiviral drugs together with CPs. Thus, there is a necessity to develop effective and safe therapeutics/drugs or vaccines against SARS-CoV-2 to control the COVID-19 spread and prevent both its recurrence and new mutation-based emergent infections. Therefore, developing NmAbs-based cocktails constitutes a quick alternative way against SARS-CoV-2 while waiting for a vaccine whose development is expensive, time-consuming, and unpredictable, as the final product does not always result in or provide a satisfactory therapeutic rate.

**FIGURE 4:**
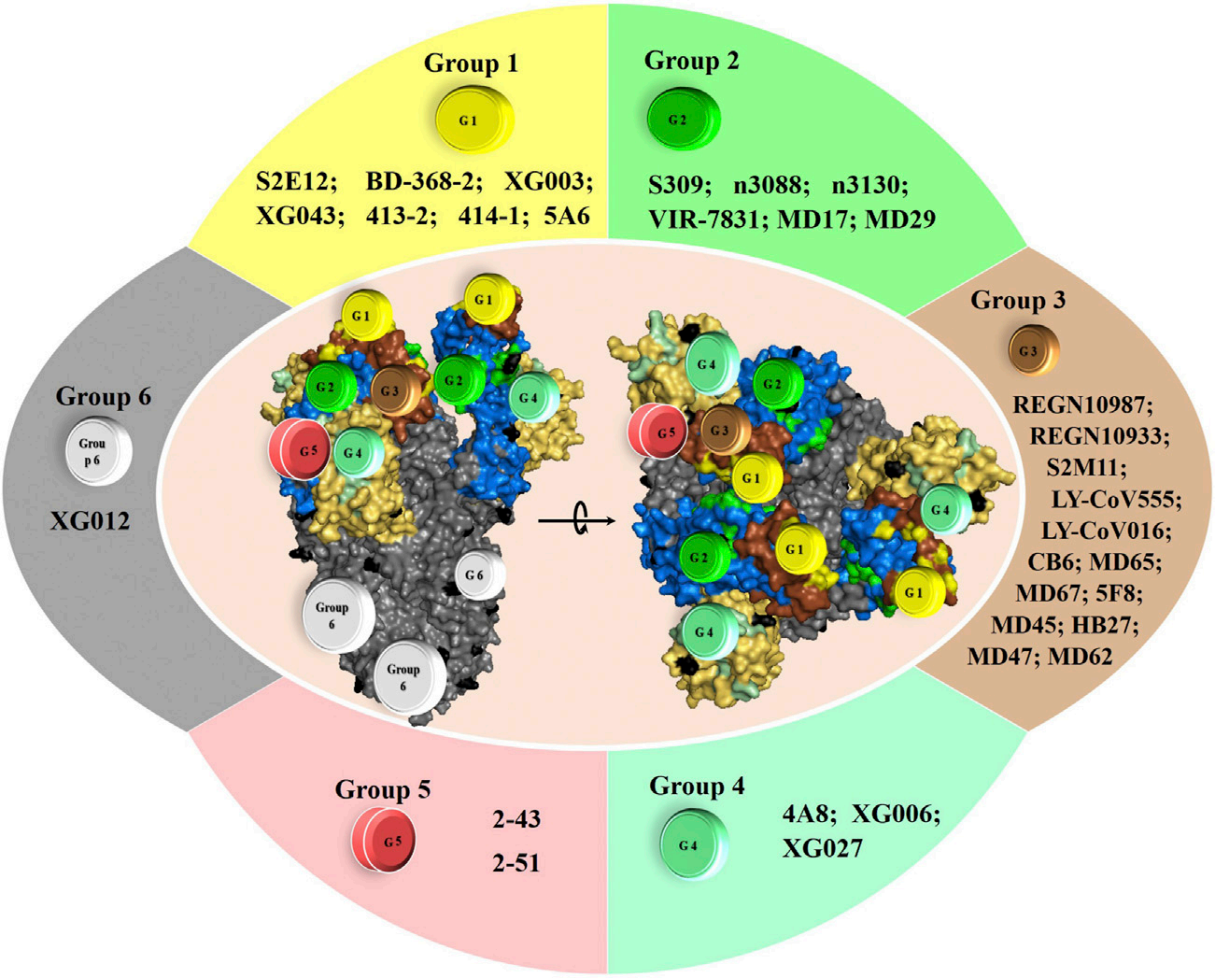
Top NmAbs in advanced clinical trial phase III and the potential NmAbs for efficient cocktail formulation. The need for potential emergency treatment for COVID-19 has birthed a dozen antibodies in clinical trials. The most advanced of them are in clinical trial phase III, and they include: i) REGN10933+REGN10987 (clinicaltrials.gov; ID: NCT04452318), sponsored by Regeneron Pharmaceuticals; ii) LY-CoV555+LY-CoV016 (LY3819253+LY3832479) (clinicaltrials.gov; ID: NCT04427501), sponsored by Eli Lilly and Company of Indianapolis; iii) LY-CoV555 (LY3819253) (clinicaltrials.gov; ID: NCT04497987), sponsored by Eli Lilly and Company of Indianapolis; iv) VIR-7831 (fully NmAb engineered from S309 features) (clinicaltrials.gov; ID: NCT04545060), sponsored by Vir biotechnology and GSK.

For instance, Hansen et al. recently showed that formulation of an antibody cocktail comprising two neutralizing antibodies, REGN10987 and REGN10933 (Hansen et al., 2020), targeting different regions on spike RBD domain exhibits a synergic neutralizing effect against both WT and two PT SARS-CoV-2, suggesting that they can be used to treat COVID-19 patients. More interestingly, they demonstrated that formulation of such antibody-based cocktails would be the key for passive immunization in reducing SARS-CoV-2 related disasters, as their formulation can prevent rapid mutational escape observed when using individual antibodies (Baum et al., 2020). Similarly, a proposed cocktail formulated with SARS-CoV-2 NmAbs S2M11 and S2E12 or S309 (Tortorici et al., 2020) proved neutralization effectivity against several SARS-CoV-2 strains. Further NmAbs are also good candidates alone or in cocktail mixture for preventing SARS-CoV-2 spread and reduce the worldwide COVID-19-associated mortality. Some are currently under consideration in clinical trials (Figure 4).

Interestingly, combining 2, 3 or 4 neutralizing monoclonal antibodies from group 1, 2, 3, 4 and/or 5 may achieve greater neutralizing rate and more potently lead to the control of SARS-CoV-2 infection, as each group of NmAbs exhibit different neutralization mechanisms (Figures 1–3 **)**. Thus, such cocktails may block the virus entry by a hypothetical enhanced synergic neutralizing effect (Figure 4), and more importantly, not leading to the development of escape mutants. Several reports supported such achievement (Elshabrawy et al., 2012; Baum et al., 2020a; Baum et al., 2020b; Hansen et al., 2020; Tortorici et al., 2020).

Tortorici *et al.* demonstrated that S2E12 from group 1 potently neutralizes SARS-CoV-2 through efficient binding to the pre-fusion S trimer, targeting residues 455-458 and 473-493 of RBM. They also revealed that S2M11, a group 3 NmAbs, neutralizes SARS-CoV-2 by targeting huge surfaces onto two neighboring RBDs within the same trimer, comprising RBM residues from one S monomer (440, 441) and RBD—but not RBM—residues from adjacent S monomer (339-343, 367-374 and 436). Thus, the S2M11 + S2E12-based cocktail could be effective, as it exhibits an additive neutralization effect and neutralizes variants that single S2M11 NmAb could not. Interestingly, it seems to us that the enhanced neutralizing effect observed for this cocktail (Tortorici et al., 2020) is probably due to the supplementary neutralizing effect of S2M11, which, however, seems not to contribute to the ability of the cocktail to neutralize some circulating mutants (including Y449N, E484K/Q, F490L, and S494P RBD variants). Indeed, S2M11 alone is unable to neutralize some mutants, a fact that is not observed for S2E12, which is effective against all tested variants. Should this fact be verified, this cocktail would potentially lead to the development of S2M11-induced escape mutants, which would be hardly observed using the S309 + S2E12-based cocktail (Tortorici et al., 2020), as S309 cross-neutralize WT and PT variants and S2E12 potently neutralize authentic and circulating variants (Pinto et al., 2020a; Pinto et al., 2020b; Tortorici et al., 2020).

Furthermore, besides the RBD-ACE2 binding, it has also been recently proven that the first step of the SARS-CoV-2 infection cycle lies on a simultaneous primary interaction of S protein with HS on the cell surface. The binding of SARS-CoV-2 RBD protein occurs directly with residues R346, R355, K444, and R466, and through hydrogen bonds with F347, S349, N354, G447, Y449, and Y451, thus stabilizes and promotes the binding of S and ACE2 (Clausen et al., 2020). However, S309, a SARS-CoV-specific group 2 NmAbs, binds to SARS-CoV-2 RBD residues 337-344 and 356-361, thus buries a surface partially involved in the RBD-HS binding; as such, it directly prevents the RBD-HS binding, explaining the neutralization efficiency of S309 NmAb. Like S309, NmAbs n3088 and n3130, two other antibodies from group 2, can be employed in the formulation of cocktails, because as nanobodies, they are stable and can potently reach their target sites more suitably and easily than full antibodies, as they are less susceptible to steric blockage, because of their small size (van der Linden et al., 1999; Dumoulin et al., 2002; Forsman et al., 2008; Laursen et al., 2018). Thus, by targeting the neutralization site away from the ACE2-binding site, they exhibit a similar mechanism as that of S309. Therefore, with this novel neutralization mechanism of group 2 NmAbs, S309 + S2E12-, n3088 + S2E12- or n3130 + S309-based cocktail would be more promising.

Moreover, a cocktail formulated from S2E12 and S309, n3088 or n3130, mixed with one or two of REGN10987, REGN10933, LY-CoV555, and LY-CoV016 from group 3, that target epitopes (G446, Y449, Q498, and T453, G484, P486, C488, Y489, respectively) on RBD different from those of group 2, and partially overlapping S2E12, might be promising as well. Indeed, by targeting almost different binding sites (Figures 1, 3, 4), they are unlikely to compete with each other for binding SARS-CoV-2 S protein. Further, REGN10987, REGN10933, S2E12, and S309 have been shown to promote activation of effector functions, including ADCC and ADCP (antibody-dependent cellular phagocytosis), which play a role in viral clearance (Baum et al., 2020b; Baum et al., 2020a; Hansen et al., 2020; Pinto et al., 2020a; Pinto et al., 2020b; Tortorici et al., 2020).

The NTD-specific hNmAbs, 4A8 deserve attention in formulating antibody-based cocktails against SARS-CoV-2. Chi et al. demonstrated that every single 4A8 targets and binds to a monomer within the trimer and burys a huge surface (141–156 and 246–260, around 832 Å^2^) at the binding interfaces and potently blocks the interaction with gangliosides justifying the neutralization potency observed against the wild-type (WT) and pseudotyped (PT) virus (Fantini et al., 2021). Therefore, a NmAbs-based cocktail comprising 4A8 would potentially block the infection. Thus, we suggest that cocktails from these highlighted NmAbs can achieve supplementary inhibition mechanisms *in-vivo* other than direct viral entry inhibition.

The spike proteins of all HCoVs consist of two subunits, S1 and S2. So far, only two antibodies targeting the S2 subunit of SARS-CoV-2 with modest and very week neutralizing activities have been reported, respectively **(** Table 3). Several antibodies against SARS-CoV were developed and showed to bind SARS-CoV S2 with high affinity (K_D_ < 10 nM). Additionally, these SARS-CoV S2-specific antibodies could bind to the trimeric spike protein with high affinity as well, exhibiting efficient therapeutic neutralizing effects, thus suggesting to confer protection against SARS-CoV (Elshabrawy et al., 2012). Therefore, because of the similarities shared by SARS-CoV S2 and SARS-CoV-2 S2 (Zheng et al., 2020), the S2 subunit of SARS-CoV-2 would be another potential neutralization target site. Developing potent neutralizing monoclonal antibodies targeting the SARS-COV-2 S2 protein may be promising in such a way that into a cocktail with other site specific-antibodies, they can promote tremendous neutralizing efficacy through the prevention of S protein fusion.

Possible treatments against SARS-CoV-2 might also consist of a NmAbs-based cocktail combined with the currently used antivirals for treating COVID-19 patients. Interestingly, Mengist et al. (Mengist et al., 2020a) highlighted the fact that the use of α-ketoamide inhibitors against SARS-CoV-2 M^pro^ efficiencily block the COVID-19 progression, and that further researches on inhibitors targeting SARS-CoV-2 main protease might constitute attracting way to tackle COVID-19 (Mengist et al., 2020a; Mengist et al., 2020b; Mohammad et al., 2020; Mengist et al., 2021). Thus, combination of these potential drugs with NmAbs might highly eradicated the pandemics due to coronavirus. Administration of CPs demonstrated an efficient protective effect during the first months of the COVID-19 outbreak (Jean et al., 2020; Kruse, 2020; Shen et al., 2020). Numerous studies reported that the CP was administered together with available antivirals in most cases, resulting in increased COVID-19 patient recovery. Therefore, NmAbs-based cocktails administered with antivirals may hypothetically drive a potent fast cure of both mild and severe COVID-19 patients, as the combination of CP with antivirals proved effective.

## Challenges

### Limit as a Biological Diagnostic Tool

All NmAbs that cross-react with SARS-Co V and SARS-CoV-2 recognize conserved epitopes, most of which are located within RBD (Tables 1, 2). The homology between sarbecovirus RBDs shows that all strains share similarities, suggesting that monoclonal antibody-based ELISA might lead to false positives for SARS-CoV-2 diagnosis because of probabilities for the test to cross-react with circulating sarbecoviruses.

### Passive Antibody Therapy

Unlike active vaccination, which induces a very long-lasting immune response (even though it takes time to be established), passive antibody immunization, though providing immediate immune effects, does not last long (less than six months depending on the antibody amount administered), suggesting a limit for the use of antibodies to protect individuals in a very long time. This limit can be paved if a well-established schedule is implemented.

### Virulence and Immune Evasion Factors

#### SARS-CoV-2 S Protein Glycan Shields

The primary steps of SARS-CoV-2 entry into the target cells occur through binding between S protein and hACE2. This recognition is more effective as the S protein's three-dimensional structure in the trimeric form is stable. Note that several viruses, including HIV, Hendra virus, influenza virus, Hepatic viruses, Nile Valley virus, and SARS-CoVs use glycosylation, a host cell post-translational modification, to modify the structure of their surface glycoproteins, which leads to the production of a more stable multimeric quaternary protein structure playing an essential role in virulence, antigenicity and evasion to the immune system (Vigerust and Shepherd, 2007). In the context of COVID-19, S protein has been shown to contain several glycans responsible for the extensive glycosylation of SARS-CoV-2 spike protein (Figure 3). These glycan-based glycosylation covers approximately 50% of the S protein epitopes, thus blocking access to recognition by known antibodies. Only an insufficient small surface area remains accessible by NmAbs (Grant et al., 2020). Besides, note that this hyper-glycosylation of SARS-CoV-2 S protein can lead to multiple conformation changes (Grant et al., 2020), suggesting limited neutralizing effects of some known NmAbs, though found to be potent neutralizers. Although the glycosylation of SARS-COV-2 protein does not occur on the hACE2 binding site (Figure 3), as RBD is the selective domain for coronavirus to survive over time, the shielding effect of glycans on SARS-CoV-2 S epitopes helps the virus to escape both the host immune system and the passive antibody therapy based immunization. This mechanism as well decreases the host potential to produce an effective immune response against SARS-CoV-2. Thus, as for HIV and other viruses, antigen glycosylation of SARS-CoV-2 S protein constitutes a potential limit point in drug/vaccine development and COVID-19 immunotherapy.

#### Molecular Mutations

On the other hand, obtaining good NmAbs and using them as monotherapetic is still not without consequences for the genetics of the virus. As with antibiotics, antivirals, including antibodies administered as monotherapy, lead to immune pressure causing mutations in the viral protein gene involved in the infection process and allowing the virus to selectively evade the adaptive immunity induced by the antibody (Baum et al., 2020b). In their study, Baum *et al.* specifically demonstrated that the development of S mutation-based SARS-CoV-2 resistance against the known potent NmAbs is provoked through treatment with both single NmAbs and antibody cocktails obtained from combinations of NmAbs targetting the same overlapping regions (Baum et al., 2020b). This suggests that NmAbs choice in formulating cocktails against SARS-CoV-2 should be taken into account for an advantage, as described previously.

## Discussion

This analytic comprehensive literature survey presents a signifying representative, but not an exhaustive, repertory of the molecularly characterized neutralizing monoclonal antibodies (NmAbs), with potentials to inhibit SARS-CoV-2 target cell entry and protect from COVID-19. Specifically, this repertory includes not only the SARS-CoV-2 specific NmAbs but also the SARS-CoV, MERS-CoV, as well as other human coronaviruses (HCoV)-specific NmAbs, presenting the main production technologies and discussing the NmAbs molecular mechanisms of actions at their specific neutralization target sites or structural epitopes. Based on the discussed molecular mechanisms of actions and, the advantages and limitations of the NmAbs use, cocktail formulations are highly suggested to mitigate COVID-19 and eventual variants.

These last decades, the world is experiencing waves of emergence and re-emergence of pathogens (bacteria or viruses), more and more difficult to contain with the current scientific technologies, thus resulting in considerable lethality rates in human and animal kingdoms. When it comes to fire viruses, including Ebola, Chikungunya, Hendra, Rabies, and lately Corona viruses, the technology of antibody-based immunotherapy (using innovative scientific technologies) constitutes the way to mitigate these eye-invisible living beings. Attractively, the development methods of NmAbs against viruses, specifically SARS-CoV-2, have seen a huge improvement. The current approaches to produce viral antibodies lie on animal immunization and convalescent plasmas through the ribosome, phage, yeast, and retained display, B cell sorting technologies (BalcioGlu et al., 2020; Beasley et al., 2020; Li T. et al., 2020; Wu et al., 2020a; Wu et al., 2020b; Zost et al., 2020b), which yield in pools of antibodies selectively potent, most of which with less somatic hypermutations, and sometimes with low levels of neutralizing activity in most convalescent plasmas (Robbiani et al., 2020). Though they are effective, these ways are laborious, sometimes incompatible for some antigens, antibodies obtained cannot be tolerated by the immune system, thus limiting their spectrum use. To pave these limitations, new technologies such as Machine Learning technologies (Magar et al., 2020), proteomics (Voss et al., 2020), artificial combination, and somatic hypermutation-based antibodies (Custódio et al., 2020; Han et al., 2020; Li T. et al., 2020; Miersch et al., 2020; Rouet et al., 2020; Rujas et al., 2020; Schoof et al., 2020; Wellner et al., 2020) have reduced the time-cost and facilitated the nowadays production of highly potent neutralizing antibodies against SARS-CoV-2. For instance, AHEAD technology is attractive for antibody development in that way that antibodies acquire some induced mutations that significantly enhanced the neutralization activity of antibodies, which can be obtained within few times (Wellner et al., 2020). Besides, synthetic approaches consisting of combining different antibody Fc-fragments yield to multivalent antibodies, with fewer possibilities to lead to antibody-based escape variants (Rujas et al., 2020), compared to standard methods. These methods are tools to overcome antibody affinity limits and develop broad and potent neutralizing antibodies, and their use may speed up the timeline from discovery to antibody production against COVID-19 (Rujas et al., 2020).

The overall neutralizing SARS-CoV-2 antibodies reported in this review only target structural epitopes on spike protein, thus confirming this glycoprotein as an excellent therapeutic target. Moreover, it comes out from this review that the reported SARS- and MERS-CoV NmAbs with cross-protection activity against SARS-CoV-2 target conserved molecular epitopes between all the coronavirus. However, because of some inherent molecular differences in cell infection, SARS-CoV or MERS-CoV specific antibodies that recognize conserved epitopes in SARS-CoV-2 might not display the same neutralization effect. That is the case of CR3022 and m396, which potently neutralize SARS-CoV but are sterically not potent to block SARS-CoV-2 infection, yet targeting conserved epitopes (Chi et al., 2020b; Yuan M. et al., 2020). Moreover, all cross-neutralizing antibodies, and almost all the SARS-CoV-2 specific NmAbs target SARS-CoV-2 RBD site, which is no more considered as the unique or the main therapeutic target site, as Voss *et al.* in their COVID-19 patients’ cohort, found that NTD represents the main site targeted by almost all NmAbs—except one which targeted RBD—that they isolated from that cohort (Voss et al., 2020). Therefore, to efficiently mitigate COVID-19 using NmAb-based immunotherapy, SARS-CoV-2 type-specific antibody approach is recommended to be adopted, specifically by considering the whole extracellular domain of spike protein, including NTD, RBD, and S2. More interestingly, the understanding of the molecular mechanism of actions summarized and deeply discussed hereinbefore is based on the targeted molecular epitopes of the characterized NmAbs, leading us to suggest some antibodies as therapeutics.

Thus, regarding the antibodies proposed in this review as potential neutralizers for cocktail formulations in order to efficiently mitigate COVID-19, several parameters have been taken into account. Specifically, and as a summary to what has been intensely discussed above, these basic parameters include but not strictly i) molecular specificity to SARS-CoV-2, ii) high single neutralization potency, iii) structural and molecular evidence of the absence of competitive neutralization between the chosen antibodies and, iv) human origin-based NmAbs.

## Conclusion

From the database screening of scientific publications, around 35 cross-neutralizing monoclonal antibodies and above 450 specific-neutralizing monoclonal antibodies against SARS-CoV-2 have been reported. They were developed from both human (healthy, COVID-19, and SARS survivors) and non-human sources (alpaca, mice, camel, equine, and llama), as well as by synthesis means, using numerous methodological approaches, from empirics to innovative. Almost all NmAbs strongly bind respectively to SARS-CoV-2 S with high affinity (K_D_ < 10 nM) and exhibit high neutralizing effects against either wild-type or pseudotyped virus, or both, confirming SARS-CoV-2 S protein as the main therapeutic target for developing drugs/vaccines.

Overall, the NmAbs revealed 5 molecular neutralization sites within SARS-CoV-2 S extracellular or ectodomain (SARS-CoV-2 S ECD), carried within 3 regions: NTD, RBD, and S2, which carry 1, 3, and 1 neutralization sites, respectively. According to these neutralization target sites, the reported NmAbs are classified into 6 groups (1 to 6), blocking the entry of SARS-CoV-2 into the cells by preventing the formation of any bounds between the SARS-CoV-2 S protein and the cell surface receptors by different neutralization mechanisms. These mechanisms include blocking through i) a direct or indirect RBD-ACE2-binding inhibition, ii) a spike protein conformation changes-based steric hindrance, iii) a blockage of RBD to adopt the “open” conformation, iv) a direct RBD-HS-binding inhibition, vi) a direct blockage of binding between NTD GBD and cell membrane lipid rafts, and vii) inhibition of the post-receptor-binding steps (virus fusion to the cell membrane).

The development of NmAbs against SARS-CoV-2 is taking a considerable place in medical research, as these products provide colossal importance in diagnostic and therapeutic follow-ups. Moreover, their use in fighting the Ebola virus results in attractive outcomes such that it is highly believed that NmAbs would lead to the eradication of COVID-19, the SARS-CoV-2 associated disease. Using specific NmAbs against SARS-CoV-2 instead of CPs highly mitigates and avoids the risk of developing ADE effects. Therefore, thanks to the presence of numerous neutralization sites, and with this repertory of SARS-CoV-2 site-specific NmAbs, formulation of cocktails, like REGN-COV-2, a mixture of REGN10987 and REGN10933, constitutes the best way to neutralize the virus at different steps of the infection cell cycle, to remedy the escape mutant development-associated issues, and thus to mitigate the COVID-19 spread. Moreover, in the hope of effective development of NmAbs based cocktails, a combination with repositioned antiviral drugs is an attractive treatment mean, as the combination of these later with CP led to satisfactory results in treating COVID-19 patients.

However, concerns related to the efficacy of NmAbs in diagnostic and in immunotherapy remain raised. Precisely, the biological diagnostic would be limited by probable false-positive COVID-19 due to cross-reaction with other *Sarbecovirus* and new emergent variants caused by the immune pressure, while the development of cocktails would be limited by the viral virulence factors such as glycosylation of spike protein and the short-term duration of the induced passive immune.
